# Postoperative pericardial effusion on routine echocardiography: A review of incidence, progression, and management: To dissolve or to be resolved

**DOI:** 10.1007/s12471-026-02054-6

**Published:** 2026-06-05

**Authors:** Stefan van Dinter, Laurens Wollersheim, Wilson Li, Rogier Donders, Niels van Royen, Hendrik-Jan Dieker, Ad Verhagen

**Affiliations:** 1 Department of Cardiothoracic Surgery, Radboud University Medical Center, Nijmegen, The Netherlands; 2 Department of Cardiothoracic Surgery, Frisius Medical Center, Leeuwarden, The Netherlands; 3 Department for Health Evidence, Section Biostatistics, Radboud Institute of Health Sciences, Radboud University Medical Center, Nijmegen, The Netherlands; 4 Department of Cardiology, Radboud University Medical Center, Nijmegen, The Netherlands

**Keywords:** Pericardial effusion, Cardiac tamponade, Cardiac surgical procedures, Echocardiography, Risk factors, Postoperative care, Postoperative complications, Pericardiocentesis

## Abstract

Postoperative pericardial effusion (PPE) is a common finding after cardiac surgery, with reported incidences ranging widely from 2% to 85%. While most effusions resolve spontaneously, a subset progresses to cardiac tamponade requiring urgent intervention. This systematic review evaluated the true incidence of PPE and PPE-related reinterventions based on routine echocardiographic screening, and explored temporal evolution, symptomatology, and perioperative risk factors. A comprehensive search of Embase, PubMed, and Web of Science through May 2025 identified 26 eligible studies including 8,495 patients. Data extraction followed PRISMA guidelines and quality assessment with the JBI checklist. Random-effects meta-analysis demonstrated a pooled PPE incidence of 36% (95% confidence interval (CI) 25–49%), whereas only 3% (95% CI 2–4%) required reintervention, ranging from 2% after CABG to 6% after aortic surgery. Large effusions (> 2 cm) were associated with a substantially higher likelihood of intervention (54% [14–90%]), although spontaneous resolution remained common. Preventive strategies such as posterior pericardiotomy and posterior chest tube placement significantly reduced both PPE incidence and reintervention rates. Despite these findings, interpretation is limited by substantial heterogeneity in study design, definitions, and follow-up protocols. Standardized definitions, structured echocardiographic follow-up, and prospective studies are needed to improve risk stratification and guide clinical decision-making.

**Supplementary Information:**

The online version of this article (10.1007/s12471-026-02054-6) contains supplementary material, which is available to authorized users.

## Introduction

**Fig. 1:**
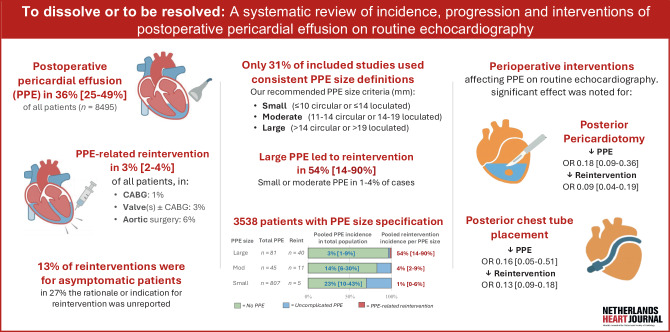
Infographic of the systematic review of incidence, progression and interventions of postoperative pericardial effusion on routine echocardiography

Cardiac surgery is a leading cause of pericardial effusion, alongside malignancy and infectious disorders [1]. Postoperative pericardial effusion (PPE) typically manifests between 48 h and over 30 days after surgery, in contrast to effusion from excessive primary postoperative hemorrhage within the first 2 days after surgery. Reported incidences range widely from 2% to 85% [2–4]. This considerable variation is influenced by perioperative factors such as surgery type and anticoagulant use, as well as by differing practices and definitions in routine postoperative echocardiography of PPE, which may include only symptomatic or also asymptomatic effusion (Fig. 1; [5–7]). Since most postoperative echocardiographic examinations are reserved for symptomatic patients or high-risk surgery, the true incidence of PPE remains debated.

PPE can progress to life-threatening cardiac tamponade requiring emergent intervention, though asymptomatic effusion may resolve spontaneously [5, 8]. Optimal management of asymptomatic PPE is still under discussion, particularly in cases of moderate severity, as highlighted by prior work reporting significant interprofessional variation in management approaches to PPE [9]. This underscores the need for research to guide optimal clinical management.

Although several studies examined PPE incidence and its natural resolution or progression to tamponade, no comprehensive literature review has analyzed the existing data [8, 10]. In this review, we summarise the incidence of postoperative pericardial effusion based on routine echocardiography, and discuss its temporal evolution, clinical relevance, and perioperative determinants. To do so, we performed a systematic search of previous literature on PubMed, Embase, and Web of Science, of which further details (framework, search strategies and criteria, literature evaluation, and statistics) are in the Electronic Supplementary Material [ESM] (Table S1–S3; Fig. S1).

## Definitions of pericardial effusion and tamponade

*PPE:* Any pericardial effusion exceeding the physiological amount, previously reported as > 50 ml or > 10 mm on echocardiography [11]. To allow pooled analysis of heterogeneous data, we used size criteria reported by the included studies and grouped them into small, moderate, and large PPE categories.

*Cardiac tamponade:* Any symptomatic PPE (e.g., dyspnea, malaise/fatigue, or chest discomfort) causing hemodynamic deterioration requiring urgent intervention [12].

*PPE-related interventions:* Any postoperative intervention to evacuate PPE, including surgical (re-sternotomy, re-thoracotomy, sub-xiphoidal drainage) and percutaneous (TTE-/CT-guided pericardiocentesis) approaches. Asymptomatic effusion evacuation was also included.

Importantly, this review focuses exclusively on sub-acute and late PPE, defined as occurring on or after postoperative day (POD) 3. This contrasts with acute PPE (< 48 h postoperatively), usually due to bleeding or coagulopathy requiring urgent re-operation [13]. The systematic review included 26 of 3,106 identified articles (See ESM Table S1), involving 8,495 patients receiving at least one routine TTE post-cardiac surgery. The average TTE timing was on POD 19.5 (median on POD 7). Patients underwent an average of 2.7 TTEs.

## Incidence of postoperative pericardial effusion

**Fig. 2:**
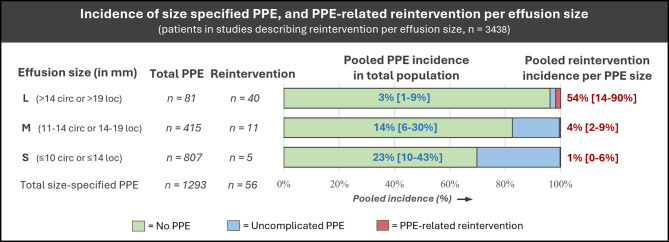
Pooled incidence of PPE and PPE-related reinterventions per effusion size. Bar charts indicating the distribution between uncomplicated PPE and PPE-related reinterventions in patients with data available on the size specification of PPE, along with the pooled incidence per size. The green bars depict the proportion of the population without PPE, blue bars with PPE without the necessity of intervention and red PPE-related reinterventions. *Circ* circular, *Loc* loculated, *L* large, *M* moderate, *PPE* postoperative pericardial effusion, *S* small

The incidence of PPE has been variably reported, largely depending on study design and echocardiographic follow-up. Incidence of PPE ranged from 6% to 85%, with a pooled incidence of 36% [25–49%] (Tab. 1; ESM Fig. S2A). Two studies (Bunge et al., Cakalagaoglu et al.) were excluded from the pooled PPE analysis due to having 100% PPE incidence (counting no PPE towards small PPE). Sixteen of 26 studies (*n* = 5,979) reported PPE incidence per surgery type: 44% [30–59%] for isolated CABG, 41% [21–66%] for valve(s) ± CABG, and 10% [7–15%] for aortic surgery (ESM Fig. S2B). In addition, 16 studies (*n* = 4,093) reported PPE sizes, and 8 studies (*n* = 3,438) reported effusion size for both PPE and PPE-related reinterventions. Small and moderate effusions were most common (23% [10–43%], 14% [6–30%] respectively), in contrast to large effusions (3% [1–9%]; Fig. 2; ESM Fig. S2C). One study combined small and moderate PPE incidence (32.5%), and three studies grouped moderate and large effusions (17.7%). One study (Shvartz et al.) reported average PPE size instead of incidence per size (5.0 mm (3.0–6.0)).

**Table 1:** Base characteristics of included studies

Study totals	Number/incidence
Patients (*n*)	8,495
– (Isolated) CABG	5,151 (60.6%)
– Valve ± CABG	2,973 (35.0%)
– Aorta	311 (3.7%)
– Other	60 (0.7%)
Total studies included (*n*)	26
*Times surgery type studied by study*	
– (Isolated) CABG	20 (77.0%)
– Valve(s) ± CABG	19 (73.1%)
– Aorta	7 (26.9%)
– Other*	6 (23.1%)
*Pooled incidence PPE* (%, [95% CI])^#^	
– Total^‡^	36% [25–49%]
– (Isolated) CABG	44% [30–59%]
– Valve(s) ± CABG	41% [21–66%]
– Aorta	10% [7–15%]
*Pooled incidence of PPE-related reinterventions* (%, [95% CI])^#^	
– Total	3% [2–4%]
– (Isolated) CABG	2% [1–3%]
– Valve(s) ± CABG	3% [2–6%]
– Aorta	6% [3–13%]

Table with the total number of patients, surgery types described by studies and the pooled incidence of PPE and PPE-related reinterventions. Pooled incidence is shown by total and per surgery when reported in the included studies

*AVR* aortic valve replacement, *CABG* coronary artery bypass grafting, *CI* confidence interval, *PPE* postoperative pericardial effusion

^#^ Corresponding forest plots of random effects meta-analyses can be found in the supplements

^‡^ Two studies (Bunge et al. 2014, Cakalagaoglu et al. 2012) with 100% PPE incidence (both studies had no ‘no PPE’ category; counting 0–10 mm of PPE as small) were excluded from the analysis

* e.g., pulmonary embolectomy, rhythm surgery

Notably, size classification/definitions used by studies varied: five studies (31%) used < 10 mm, 10–20 mm, or > 20 mm on echocardiography for ‘small’, ‘moderate’, or ‘large’ PPE, respectively. Two studies (13%) used < 10, 10–15, and > 15 mm. One (6%) used < 10 mm posterior, > 10 posterior, and > 10 posterior and anterior. Four (25%) distinguished circumferential from loculated PPE, and two (13%) specified PPE by posterior, anterior, apical, lateral, or circumferential localization. Two (13%) studies reported PPE sizes yet lacked size definitions. Tab. 2 provides an overview of the various size criteria used in previous literature.

**Table 2:** PPE size criteria used by included studies

PPE size criteria used by studies with size specification (*n* = 16)				Number of studies (%)
Small	Moderate		Large	
< 10 mm	10–20 mm		> 20 mm	5 (31)
≤ 10 mm	11–15 mm		> 15 mm	2 (13)
≤ 10 mm posterior	> 10 mm posterior		> 10 mm posterior and anterior	1 (6)
Loculated 10–14 mm, or Circ < 10 mm	Loculated 15–19 mm, or Circ 10–14 mm		Loculated > 19 mm, or Circ > 14 mm	1 (6)
Grade 1:^*^ Loculated < 10 mm	*Grade 2:* Loculated 10–14 mm, Circ ≤ 14 mm	*Grade 3:* Loculated 15–19 mm Circ 10–14 mm	*Grade 4:* Loculated > 19 mm Circ > 14 mm	2 (13)
Loculated < 5 mm, or Diffuse (sum anterior + posterior) 1–9 mm	Loculated 5–9 mm, or Diffuse 10–19 mm		Loculated ≥ 10 mm, or Diffuse ≥ 20 mm	1 (6)
Only slight effusion, located posteriorly, or below AV-groove	Effusion more uniformly distributed, both posteriorly and apically		Effusion uniformly distributed both posteriorly, anteriorly and apicolaterally	2 (13)
Small, moderate and large sizes were used, yet no definition or cut-off values were described in the study				2 (13)

Table with all size criteria used in the included studies, along with the number of studies using the criteria. Two studies used size criteria yet did not elaborate their used criteria

*AV* atrioventricular, *Circ* circumferential, *PPE* postoperative pericardial effusion

* Meurin et al. 2004 and Christiansen et al. 2013 used grade 1–4 classification instead of a small, moderate and large classification. For our analysis, grade 1 was counted towards small, grade 2–3 to moderate and 4 to large

## Incidence and timing of PPE-related reinterventions

Pooled reintervention rate from all studies describing PPE-related reinterventions (*n* = 6,378) was 3% [2–4%], with a prediction interval of 1–9% (Tab. 1; ESM Fig. S3A). Regarding studies reporting PPE size at the moment of reintervention (*n* = 3,438), pooled reintervention rates (of total PPE) were 1% [0–6%], 4% [2–9%], and 54% [14–90%] for small, moderate, and large effusions, respectively (ESM Fig. 3C). One study combining small and moderate PPE reported no reinterventions, and two studies with combined moderate/large effusions reported 19.2% reintervention rate.

Seventeen of 26 studies (*n* = 5,190) provided PPE-related reintervention rates per surgery type, being 2% [1–3%], 3% [2–6%], and 6% [3–13%] for CABG, valve(s) ± CABG, and aortic surgery (Tab. 1; ESM Fig. S3B). Nineteen studies (83 reinterventions; 62.4% of total) noted the timing of reintervention. Of these interventions, 59% occurred ≤ 30 days post-surgery and 41% after POD 30 (ESM Table S5). CABG-related reinterventions mostly occurred after POD 30 (68%), whereas 71% of reinterventions after valve(s) ± CABG were within 30 days.

## Evolution of PPE

**Fig. 3:**
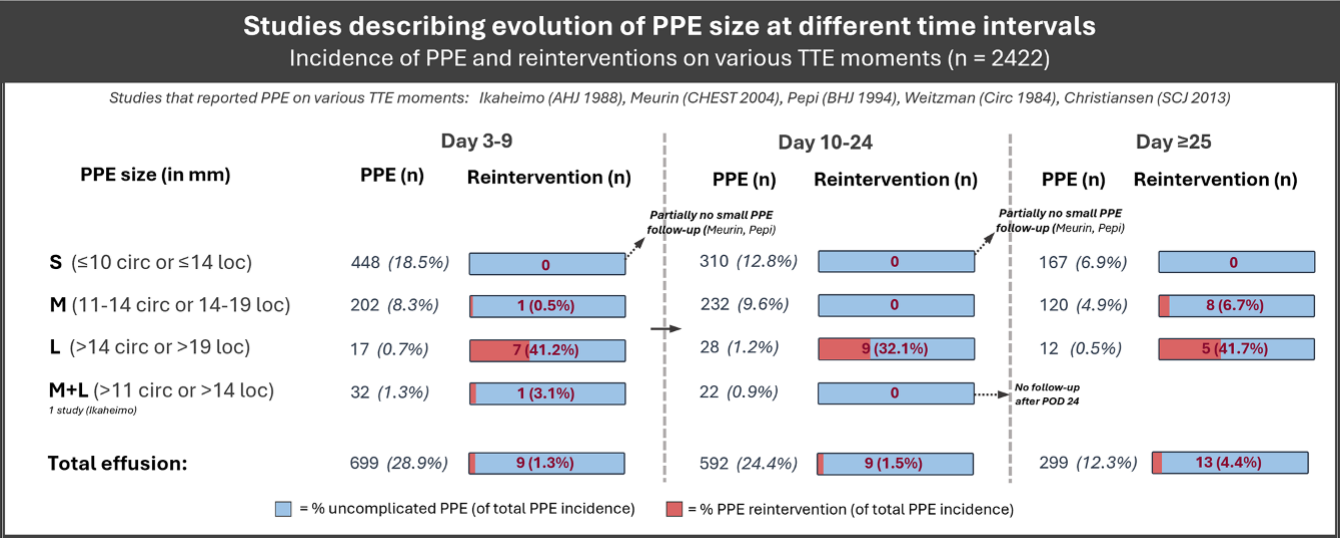
Studies describing the evolution of PPE at different time intervals. Figure showing the incidence and evolution of PPE, measured on various postoperative moments (day 3–9, 10–24, and ≥ 25). The presented studies had both time and size specified for PPE and PPE-related reintervention incidence, at least two or more TTE examinations, and no specific perioperative interventions influencing the natural course of PPE. One study (Ikaheimo et al.) combined moderate and large PPE. The blue bars display the incidence of uncomplicated PPE, red bars show the PPE-related reinterventions relative to total PPE incidence, per effusion size, and in total. *L* large, *M* moderate, *PPE* postoperative pericardial effusion, *S* small, *TTE* transthoracic echocardiogram

The temporal course of PPE provides important insight into its clinical relevance. Five studies (*n* = 2,422) assessed longitudinal evolution of PPE size and reintervention at a minimum of two distinct postoperative periods, POD 3–9, POD 10–24, and POD ≥ 25 (Fig. 3). One study (Ikaheimo et al.) combined moderate and large effusions. Among these studies, PPE was observed most on POD 3–9 (29%), mainly small- (19%) or moderate-sized (8%). Incidence of PPE gradually dropped to 24% on POD 10–24 and 12% on POD ≥ 25. Small PPE declined steadily after POD 3–9 without reported reintervention, although two studies (Meurin and Pepi et al.) discarded small PPE from further follow-up. Both moderate and large effusion incidences peaked at POD 10–24 (10% and 1%) and halved after POD 25, whereas the rate of PPE-related reinterventions increased over time from 1.3% (*n* = 9) on POD 3–9 to 4.4% (*n* = 13) on POD ≥ 25. Overall, 60% of large effusions required no intervention. Data on symptomatology at the time of reintervention were available in 97 of 133 (73%) reinterventions, while in 27% (*n* = 36) symptomatology was unreported (ESM Fig. S4). In 13% (*n* = 17) PPE was evacuated in asymptomatic patients, all having at least moderate effusion. For the remaining 60%, reintervention was performed due to symptomatic effusion.

## Interventions affecting PPE and reintervention incidence

Several studies examined perioperative strategies to reduce PPE or PPE-related reinterventions (ESM Table S6). Multiple randomized studies report significantly lower PPE incidences and no PPE-related reintervention when performing a posterior pericardiotomy with pleural drainage or retrocardiac chest tube placement. Two of the three studies examining perioperative administration of nonsteroidal anti-inflammatory drugs (NSAIDs) observed a significant reduction in PPE: one with indomethacin 7 days preoperatively and 6 weeks postoperatively (PPE from 18% to 2%, *p* 0.019), the other with ibuprofen for patients with post-pericardiotomy syndrome (86% to 50%, *p* 0.008) [14, 15]. Other factors (e.g., graft type (LIMA/vein), intraoperative dose of dexamethasone, active clearance chest tubes or prolonged drainage) showed no significant effect on PPE or reintervention incidence [16–19].

**Fig. 4:**
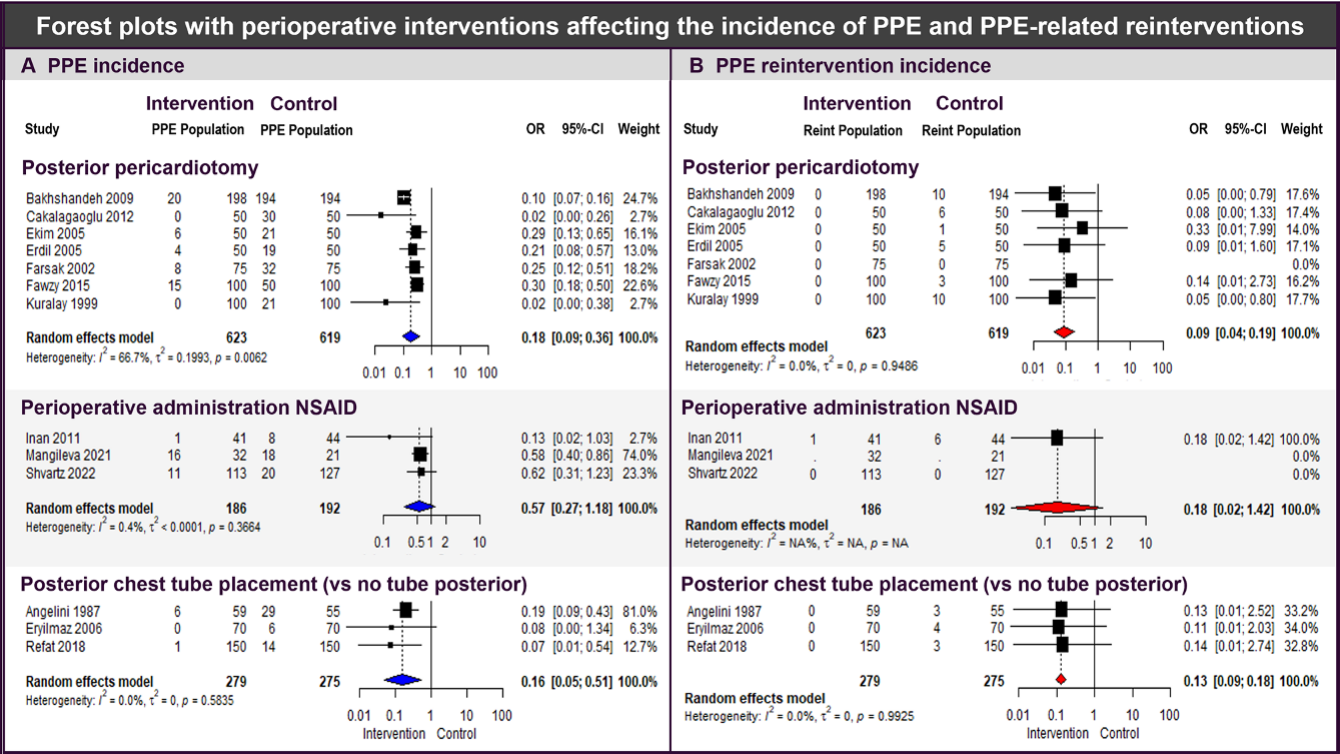
Forest plots with perioperative interventions affecting the incidence of PPE and PPE-related reinterventions. Figure showing meta-analyses with the Cochrane-Mantel-Haenszel method with corresponding forest plots of the effect of perioperative interventions on the incidence of PPE (**a**) and PPE-related reinterventions (**b**). Meta-analyses were only performed if a perioperative intervention or factor was described by multiple studies (other interventions that were described once can be found in ESM Table S5). *NSAID* nonsteroidal anti-inflammatory drug, *PPE* postoperative pericardial effusion, *Reint* reintervention

Meta-analyses with corresponding forest plots of studies examining similar interventions are shown in Fig. 4. Significantly reduced odds ratios for PPE and PPE-related reinterventions were observed with posterior pericardiotomy (OR 0.18 [0.09–0.36] and 0.09 [0.04–0.19], respectively), as well as posterior chest tube placement (OR 0.16 [0.05–0.51] and OR 0.13 [0.09–0.18]). The pooled odds ratio for Perioperative NSAID administration was not significant, although cohort sizes were limited.

## Discussion

This systematic review of 26 studies, including 8,495 patients, reports a pooled PPE incidence of 36% on routine echocardiography post-cardiac surgery and PPE-related reinterventions in 3%. For large effusions (≥ 15 mm circumferential or ≥ 20 mm loculated), PPE-related reinterventions are performed in 54%. Most reinterventions occur within 30 days post-surgery, though 42% occured after 30 days, predominantly following CABG surgery. Reported reintervention rates are highest after aortic surgery and lowest after isolated CABG, despite PPE in general being most frequent after CABG. The true incidence of cardiac tamponade was likely lower than the reintervention incidence, as only 60% of reinterventions involved symptomatic PPE. In the remaining 40%, a clear indication for reintervention or symptom documentation was lacking, or the patient was asymptomatic.

### Heterogeneity of estimated incidences

In particular, after CABG and valve(s) ± CABG surgery, the heterogeneity of pooled incidences was high, mostly exceeding an I^2^ of 75%. Consequently, study weights in the random-effects model were relatively evenly distributed, as weighting is influenced by both within- and between-study variance. This is likely to relate to varying echocardiographic PPE size criteria and timing of TTE follow-up, ranging from 1–2 weeks to several months postoperatively, limiting the interpretability of pooled estimates. Heterogeneity for aortic surgery was considerably lower (I^2^ 0% and 29% for PPE and reintervention, respectively). Similarly low heterogeneity was observed in studies reporting reintervention rates after posterior pericardiotomy. Although this may suggest a more homogeneous effect, generalizability remains limited given the small number of studies and low event counts. Nevertheless, these findings emphasize the imbalance in surgical populations studied and the need for consistent diagnostic criteria and data presentation.

### Non-uniform PPE criteria

Reported PPE incidences have ranged from 2% to 85%, due to largely varying practices in PPE definitions, routine postoperative echocardiographic evaluation, and differing cut-off criteria [5, 7]. By including only studies routinely assessing PPE, we estimate an incidence of 36%. Diagnostic and categorizing PPE criteria varied widely, with only 31% of studies using consistent definitions. We report on some studies using quantitative measurements, while others employ descriptive terms (e.g., ‘posteriorly’ or ‘uniformly’). Given contemporary echocardiographic utilities, standardized metric classifications are preferable, improving translatability and comparability. ESC guidelines recommend classifying PPE as < 10 mm, 10–20 mm, and > 20 mm for small, moderate, and large effusions, respectively [13]. We propose refining these criteria to account for loculated versus circumferential distribution, considering the reported increased tamponade risk for circumferential effusion [4, 8, 20]. Therefore, we suggest categorizing PPE as < 10 mm (small), 10–14 circumferential or 10–19 mm loculated (moderate), and ≥ 15 circumferential or ≥ 20 mm loculated (large), measured during diastole, allowing straightforward classification and comparison. By pooling size categories of the included studies, we observe a 54% reintervention rate for large PPE. Active TTE follow-up of large PPE may therefore represent a pragmatic approach to monitoring the effusion, although approximately half resolve without intervention, supporting conservative management in clinically stable and asymptomatic patients. These hypothesized thresholds warrant validation in future prospective studies, integrating effusion size, symptoms, and hemodynamic relevance. Improved documentation of rationale for PPE-related reintervention is therefore essential, as they are currently documented in only 60%.

### PPE evolution and regression

Six studies assessed PPE evolution using multiple routine echocardiograms. None report reinterventions for small PPE, supporting limited echocardiographic follow-up. However, moderate to large effusions may necessitate reintervention, even after POD 25 [7, 8, 10, 20–22], although several studies also report spontaneous resolution of these effusions, which we also show in Fig. 3 [5, 8, 23, 24]. The pericardium can both produce and reabsorb fluid physiologically, but when accumulation exceeds resorption, hemodynamic complications can arise [25, 26]. Identified risk factors for tamponade include non-CABG surgery, anticoagulant use, or platelet and erythrocyte transfusions, although mechanisms behind this imbalance between late PPE accumulation and resorption remain unclear [27, 28]. Notably, we found 42% of reinterventions occurring after POD 30, particularly after CABG. One explanation may be the Dual Antiplatelet Therapy (DAPT) in CABG patients with acute coronary syndrome, possibly increasing perioperative hemorrhage risk, contributing to additional hematoma and consequent late PPE formation [29, 30]. However, conflicting data exist, with some studies observing no tamponade difference between DAPT and aspirin users [31], while aspirin may even reduce subacute-late tamponade development due to anti-inflammatory effects [27, 28, 32, 33]. Further research is needed to clarify these associations.

### Prophylactic interventions

Several studies evaluated PPE-reducing interventions, particularly posterior pericardiotomy. These consistently show significant reductions in PPE and PPE-related reinterventions, in line with prior studies investigating posterior pericardiotomy to prevent atrial fibrillation [34, 35]. Despite our findings, these studies often focus on supraventricular arrhythmias rather than PPE, applying broad exclusion criteria (e.g., renal dysfunction, chronic obstructive pulmonary disease, beta-blocker usage), limiting generalizability. Moreover, they lack control groups with posterior or intrapericardial chest tubes due to the theoretical risk of triggering arrhythmias. This can affect PPE incidence, as we have also presented the benefits of posterior pericardial drainage [18, 36–38]. Despite this limitation, posterior pericardiotomy and other interventions such as perioperative NSAID administration may serve as additional safety measures for patients at risk for PPE progression. Additional trials to examine the prophylactic effect of these interventions in high-risk patients for PPE development could be of value.

### Research gaps

This review was subjected to the following research gaps:

*Variable diagnostic criteria and data availability:* Study populations were very heterogeneous, and data collection, including effusion size and timing of reintervention, depended on study-specific PPE definitions and measurements, contributing to heterogeneity. Although most data could be grouped into three size categories, analyses remained limited by broad confidence intervals and high heterogeneity. Also, the lack of stratification by both surgery type and effusion size disallowed analysis of PPE size per surgery type.

*Interprofessional variation:* Interprofessional variation in echocardiography measurements and interpretation is expected. However, as experienced cardiologists or cardiac sonographers conducted the examinations, a certain level of credibility and reliable effusion measurement is ensured.

*Aortic surgery:* Compared to isolated CABG (5,151) and valve(s) ± CABG (2,973) surgery, the number of aortic surgery patients (311) was small, limiting generalizability, although creating an incentive for further investigation of PPE development after aortic surgery, especially considering the increased PPE-related reintervention rate post-aortic surgery [5, 12].

*Unreported thrombi:* Intrapericardial thrombi were not reported, despite their association with reinterventions, particularly if located near the right atrium/ventricle [39]. Considering only one study reported reinterventions for small PPE, it is plausible that these cases involved small but unreported thrombi [4].

Despite these impediments, this is the first review focusing exclusively on routine echocardiography after cardiac surgery. Although impacted by the varying study methodologies and data heterogeneity, it provides a more accurate estimate of PPE incidence independent of clinical indication for TTE examination. Based on our findings, follow-up TTE is recommended for moderate to large PPE (10–14 mm circumferential or 10–19 mm loculated). Evacuating large effusions (≥ 15 mm circumferential or ≥ 20 mm loculated) may be considered, since large PPE led to reintervention in ~50%. However, the symptomatic and hemodynamic course should play a key role in this decision, and conservative management can be appropriate in stable, asymptomatic patients. Future large cohort studies should aim to identify risk factors for tamponade development and guide individualized management using patient-specific risk profiles for tamponade development. Current guidelines (ESC/EACTS and ACC/AHA/SCAI) do not address PPE and PPE-related reintervention rates, nor preventive strategies, and future guideline updates should incorporate these findings to encourage research and innovation [40–43].

## Conclusion

This review indicates a 36% incidence of PPE after cardiac surgery, with approximately 3% of patients requiring PPE-related reinterventions, of which 41% occur after postoperative day 30. Large effusions lead to reintervention in 54% of cases, in both asymptomatic and symptomatic patients, while the remainder were managed conservatively. Despite numerous reported interventions to prevent PPE, a clear explanation for why some patients develop late PPE-related complications remains elusive, and a definitive preventive strategy has yet to be identified. Nonetheless, the generalizability of our findings is limited by substantial study heterogeneity in diagnostic criteria, follow-up, and study populations. Future prospective studies are therefore essential, incorporating serial routine echocardiography, standardized PPE measurement criteria, and detailed documentation of symptomatology and risk factors. Such efforts will improve understanding of PPE evolution and support the development of clear, evidence-based guidelines and recommendations on PPE management to optimize management and reduce patient care variability.

## Supplementary Information

ESM1: Supplementary material 1

ESM2: Supplementary material 2

## Data Availability

All data supporting the findings of this work are included in the article.
