# Supplementary material for: Postoperative pericardial effusion on routine echocardiography: A review of incidence, progression, and management: To dissolve or to be resolved
Source: Neth Heart J. 2026 Jun 5;34(7-8):254–64. doi: 10.1007/s12471-026-02054-6 (PMC13375986; doi:10.1007/s12471-026-02054-6)
Supplement: Supplementary file 1 — ESM1: Supplementary material 1 [file 12471_2026_2054_MOESM1_ESM.docx]

**Electronic Supplementary Material**

**Supplementary text**

### **Search strategy, literature assessment and statistics**

This study was conducted in accordance with the PRISMA 2020 statement, following its checklist[44]. Before initiating the search, PROSPERO and the Cochrane database were reviewed to avoid duplication. The study was then registered in PROSPERO (ID CRD42023469533).

### **Research questions**

*Primary research questions:*

- What is the total incidence of sub-acute/late PPE on routine postoperative echocardiography among adult cardiac surgery patients?
- What is the total incidence of PPE-related reinterventions in adult cardiac surgery patients?

*Secondary research questions:*

- What is the relationship between PPE size and PPE-related reinterventions?
- How does PPE size develop over time in patients with established PPE undergoing multiple echocardiograms?
- How frequently are symptoms documented at the time of PPE-related reinterventions?

Do specific perioperative factors influence the PPE incidence on routine echocardiography post-cardiac surgery?

### **Assessment of literature**

Searches were conducted in Embase (Ovid), Medline (PubMed), and Web of Science (Clarivate). A PECO(S) framework guided the overall search strategy, with database-specific searches developed in collaboration with a university librarian. Two authors (SD, LW) independently screened titles and abstracts; conflicts were resolved by a third (WL). Original studies (excluding case reports) reporting PPE incidence through echocardiographic examination after adult cardiac surgery were included. After full-text review, studies were retained in which the entire cohort received routine postoperative echocardiography for PPE assessment. Further details (framework, search strategies and criteria, literature evaluation) are in the Electronic Supplementary Material [ESM] (Table S1 and S2, Fig. S1). The last search was on May 1^st^, 2025.

*Risk of bias (quality) assessment*

Study quality was assessed using the Joanna Briggs Institute Critical Appraisal Checklist for Prevalence Studies[45]. Studies scoring ≥ 7/9 (≥ 5/7 if no intervention was studied) were considered low risk of bias. Studies scoring ≤ 6/9 (or ≤ 4/7) were specifically evaluated for data credibility and utility. Details of the assessment are available in the ESM (Table S3).

### **Data management, extraction, and analysis**

*Rayyan.ai* was used for screening, and duplicates were removed via *www.dedupendnote.nl*[46, 47]. Microsoft Excel was used for data storage, and statistical analyses were performed in R[48].

Surgery types were categorized as isolated CABG, valve(s)±CABG, Aorta, or Other. PPE sizes were classified as small, moderate, or large according to study methods. Given the clinical and methodological heterogeneity between included studies, random effects meta-analysis with logit transformation to stabilize variances was performed to calculate the pooled PPE and PPE-related reintervention incidence. To pool the effect (odds ratios) of perioperative interventions on PPE and reintervention incidence, a Cochrane-Mantel-Haenszel meta-analysis was used. Results are presented with 95% confidence intervals (CI) in forest plots, and p-values < 0.05 were considered significant. Study heterogeneity was evaluated according to the Cochrane Handbook Guidance (I^2^: 25% low, 50% moderate, 75% high heterogeneity)[49]. Support was granted by a statistician (author RD) at Radboud University Medical Center.

**Supplementary references**

*References of included articles used for statistics and pooled analyses, not used for writing (see table 4 with all included articles)*

44. Shamseer L, Moher D, Clarke M, et al. Preferred reporting items for systematic review and meta-analysis protocols (PRISMA-P) 2015: elaboration and explanation. *BMJ*. 2015;349:g7647. doi: <https://doi.org/10.1136/bmj.g7647>

45. Institute JB. Critical Appraisal tools for use in JBI Systematic Reviews - Checklist for prevalence studies. 2020.

46. Lobbestael G. DedupEndNote (Version 1.0.0). 2023.

47. Ouzzani M, Hammady H, Fedorowicz Z, Elmagarmid A. Rayyan—a web and mobile app for systematic reviews. *Syst Rev*. 2016;5(1):210. doi: <https://doi.org/10.1186/s13643-016-0384-4>

48. Team RC. R: A Language and Environment for Statistical Computing (<https://www.R-project.org/>). 2025.

49. Akl E, Altman D, Aluko P, et al. Cochrane Handbook for Systematic Reviews of Interventions. 2019.
50. Shvartz V, Le T, Enginoev S, Sokolskaya M, et al. Colchicine in Cardiac Surgery: The COCS Randomized Clinical Trial. *J Cardiovasc Dev Dis*. 2022 Oct 20;9(10):363. <https://doi.org/10.3390/jcdd9100363>

51. Alsaddique A, Canty D, Fouda M, Royse C, Royse A. Impact of routine repeated transthoracic echocardiography and lung ultrasound monitoring after cardiac surgery (ITRACS) - A prospective observational study in 91 patients. *Heart Lung Circ*. 2015;24(Supplement 1):e60-e1. doi: <https://doi.org/10.1016/j.hlc.2014.12.127>

52. Bakhshandeh AR, Salehi M, Radmehr H, Sattarzadeh R, Nasr AR, Sadeghpour AH. Postoperative pericardial effusion and posterior pericardiotomy: Related? *Asian Cardiovasc Thorac Ann*. 2009;17(5):477-9. doi: <https://doi.org/10.1177/0218492309341787>

53. Cakalagaoglu C, Koksal C, Baysal A, et al. The use of posterior pericardiotomy technique to prevent postoperative pericardial effusion in cardiac surgery. *Heart Surg Forum*. 2012;15(2):E84-E9. doi: <https://doi.org/10.1532/HSF98.20111128>

54. Ekim H, Kutay V, Hazar A, Akbayrak H, Başel H, Tuncer M. Effects of posterior pericardiotomy on the incidence of pericardial effusion and atrial fibrillation after coronary revascularization. *Med Sci Monit*. 2006;12(10):CR431-4. doi: <https://doi.org/10.1111/j.1540-8191.2005.200375.x>

55. Erdil N, Nisanoglu V, Kosar F, Erdil FA, Cihan HB, Battaloglu B. Effect of Posterior Pericardiotomy on Early and Late Pericardial Effusion After Valve Replacement. *J Car Surg*. 2005;0(0):70111054008003-070111054008003. doi: <https://doi.org/10.1111/j.0886-0440.2005.200375.x>

56. Farsak B, Gunaydin S, Tokmakoglu H, Kandemir O, Yorgancioglu C, Zorlutuna Y. Posterior pericardiotomy reduces the incidence of supra-ventricular arrhythmias and pericardial effusion after coronary artery bypass grafting. *Eur J Cardiothorac Surg*. 2002;22(2):278-81. doi: <https://doi.org/10.1016/s1010-7940(02)00259-2>

57. Fawzy H, Elatafy E, Elkassas M, Elsarawy E, Morsy A, Fawzy A. Can posterior pericardiotomy reduce the incidence of postoperative atrial fibrillation after coronary artery bypass grafting?†. *Interact Cardiovasc Thorac Surg*. 2015;21(4):488-91. doi: <https://doi.org/10.1093/icvts/ivv190>

58. Kuralay E, Özal E, Demirkiliç U, Tatar H. Effect of posterior pericardiotomy on postoperative supraventricular arrhythmias and late pericardial effusion (posterior pericardiotomy). *J Thorac Cardiovasc Surg*. 1999;118(3):492-5. doi: <https://doi.org/10.1016/s0022-5223(99)70187-x>

59. Stevenson LW, Child JS, Laks H, Kern L. Incidence and significance of early pericardial effusions after cardiac surgery. *Am J Cardiol*. 1984;54(7):848-51. doi: <https://doi.org/10.1016/s0002-9149(84)80219-2>

**Supplementary figures**

Supplementary figure 1. PRISMA 2020 flow diagram with in- and exclusion of studies

Supplementary figures 2. Plots with PPE total estimated incidence, per study and per effusion size

Supplementary figure 2A. Plot with incidence of PPE per study and total estimated incidence

Supplementary figure 2B. Plots with PPE incidence per type of surgery per study, and total estimated incidence

Supplementary figure 2C. Plots with PPE incidence per effusion size per study, and total estimated incidence

Supplementary figures 3. Plots with PPE-related reinterventions total estimated incidence, per surgery type and per effusion size

Supplementary figure 3A. Plot with incidence of PPE-related reinterventions per study and total estimated incidence

Supplementary figure 3B. Plots with PPE-related reintervention incidence per type of surgery per study, and total estimated incidence

Supplementary figure 3C. Plots with PPE-related reintervention incidence per effusion size per study, and total estimated incidence

Supplementary figure 4. Reported symptomatology of PPE-related reinterventions

**Supplementary figure** **1**. PRISMA 2020 flow diagram with in- and exclusion of studies

Studies included in review

(n = 26)

Reports of included studies

(n = 26)

**Identification of studies via databases and registers (May, 2025)**

**Screening**

Records screened (title/abstract)

(n = 3106)

Records excluded based on title/abstract

(n = 3017)

Reports sought for retrieval

(n = 89)

Reports not retrieved / no full-text

(n = 17)

Reports assessed for eligibility

(n = 72)

**Included**

**Identification**

Records identified from:

Databases (n = 3.655)

PubMed (n = 696)

Embase (n = 2352)

Web of Science (n = 607)

Registers (n = 0)

Records removed *before screening*:

Duplicate records removed (n = 549)

Records marked as ineligible by automation tools (n = 0)

Records removed for other reasons (n = 0)

Reports excluded:

No English language (n = 1)

No original research (n = 2)

No cardiac surgery (n = 7)

No incidence of PPE described (n = 11)

TTE was not performed on a routine basis (n = 25)

**Supplementary figure 1.** Process of study in- and exclusion shown in the PRISMA 2020 flow diagram. In total, 26 studies were included in the review. Search was initially performed in October 2023 and repeated in July 2024 and May 2025.

TTE: transthoracic echocardiography; PPE: postoperative pericardial effusion

**Supplementary figures 2**. Plots with PPE total estimated incidence, per study and per effusion size

#### **Supplementary figure 2A**. Plot with incidence of PPE per study and total estimated incidence


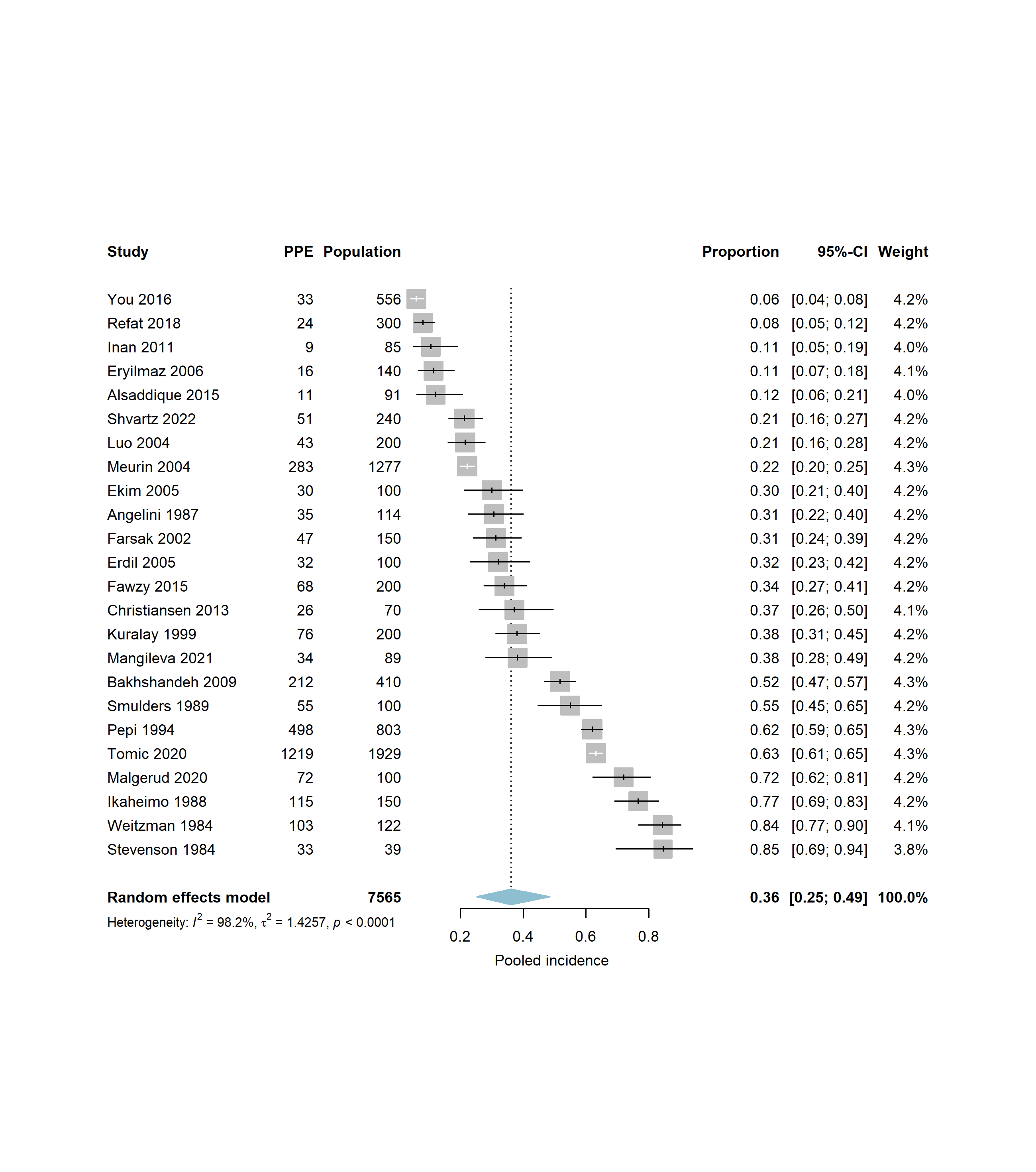


**Supplementary figure 2A.** Plot with random effect meta-analyses providing pooled incidence of routinely assessed PPE per study with 95% confidence intervals, and total estimated incidence. Two studies (Bunge et al. 2014, Cakalagaoglu et al. 2012) with 100% PPE incidence (both studies had no ‘no PPE’ category; counting 0-10 mm of PPE as small) were excluded from the total PPE pooled incidence.

#### **Supplementary figure 2B.** Plots with PPE incidence per type of surgery per study, and total estimated incidence


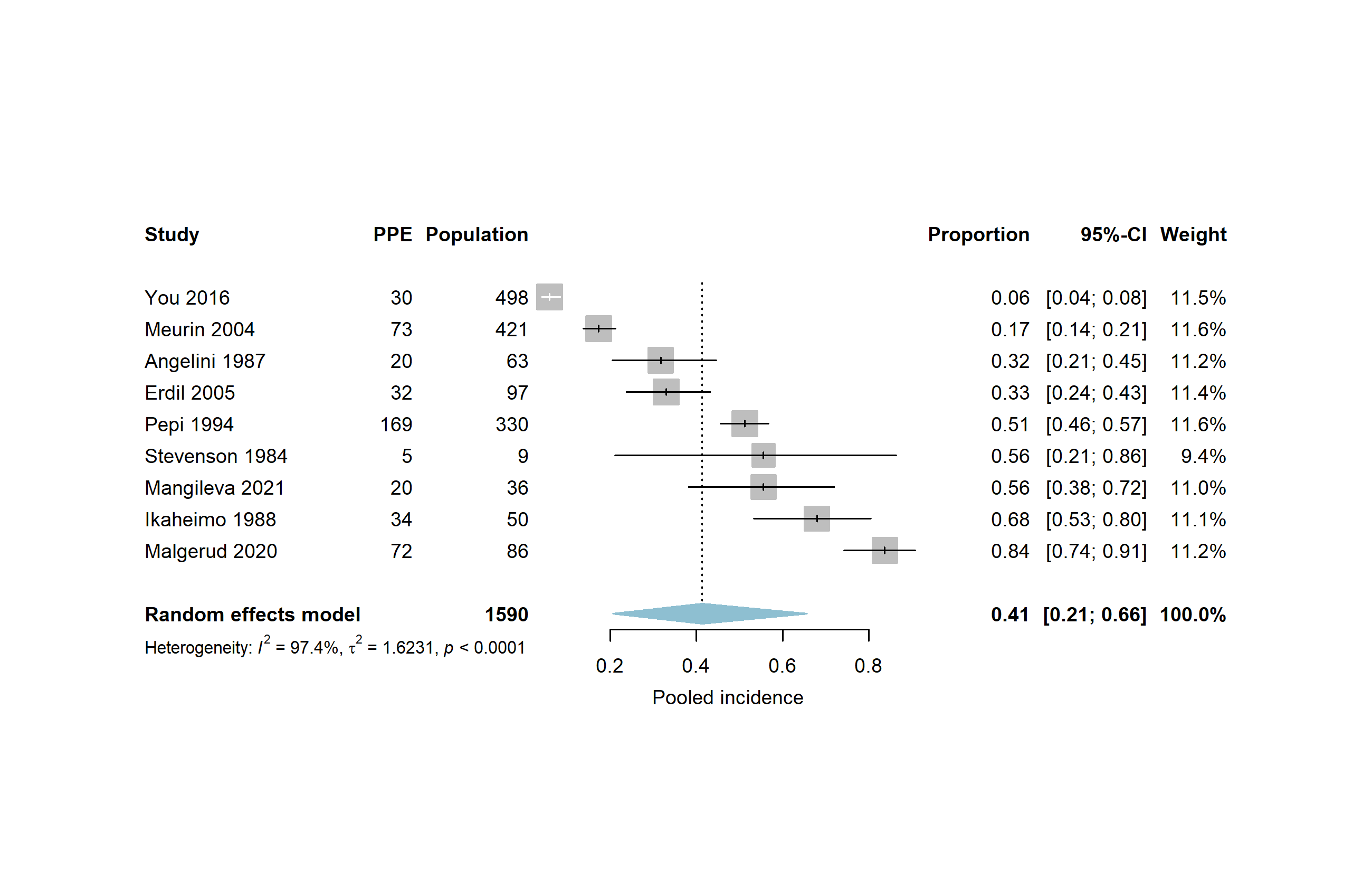

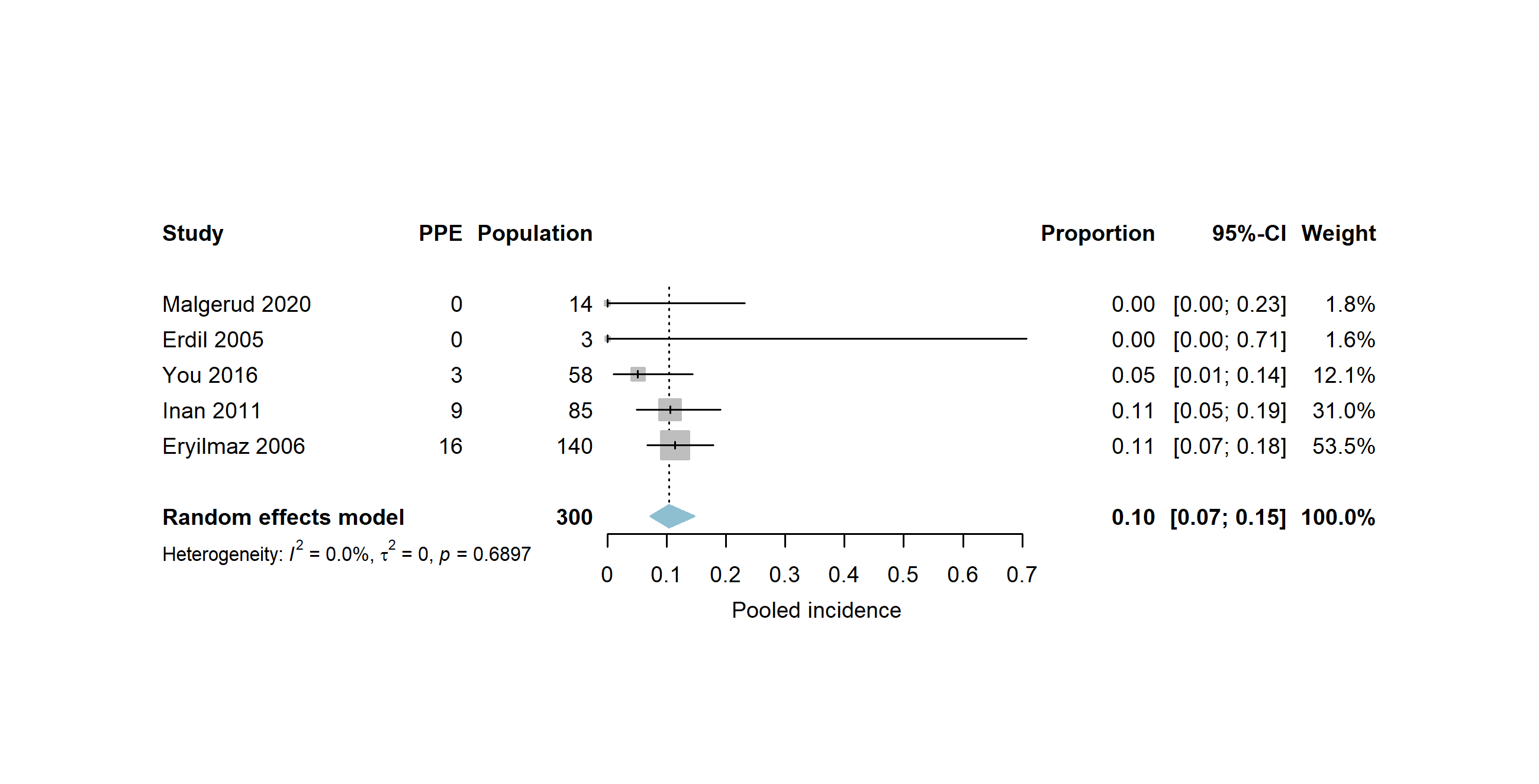


**Aorta**


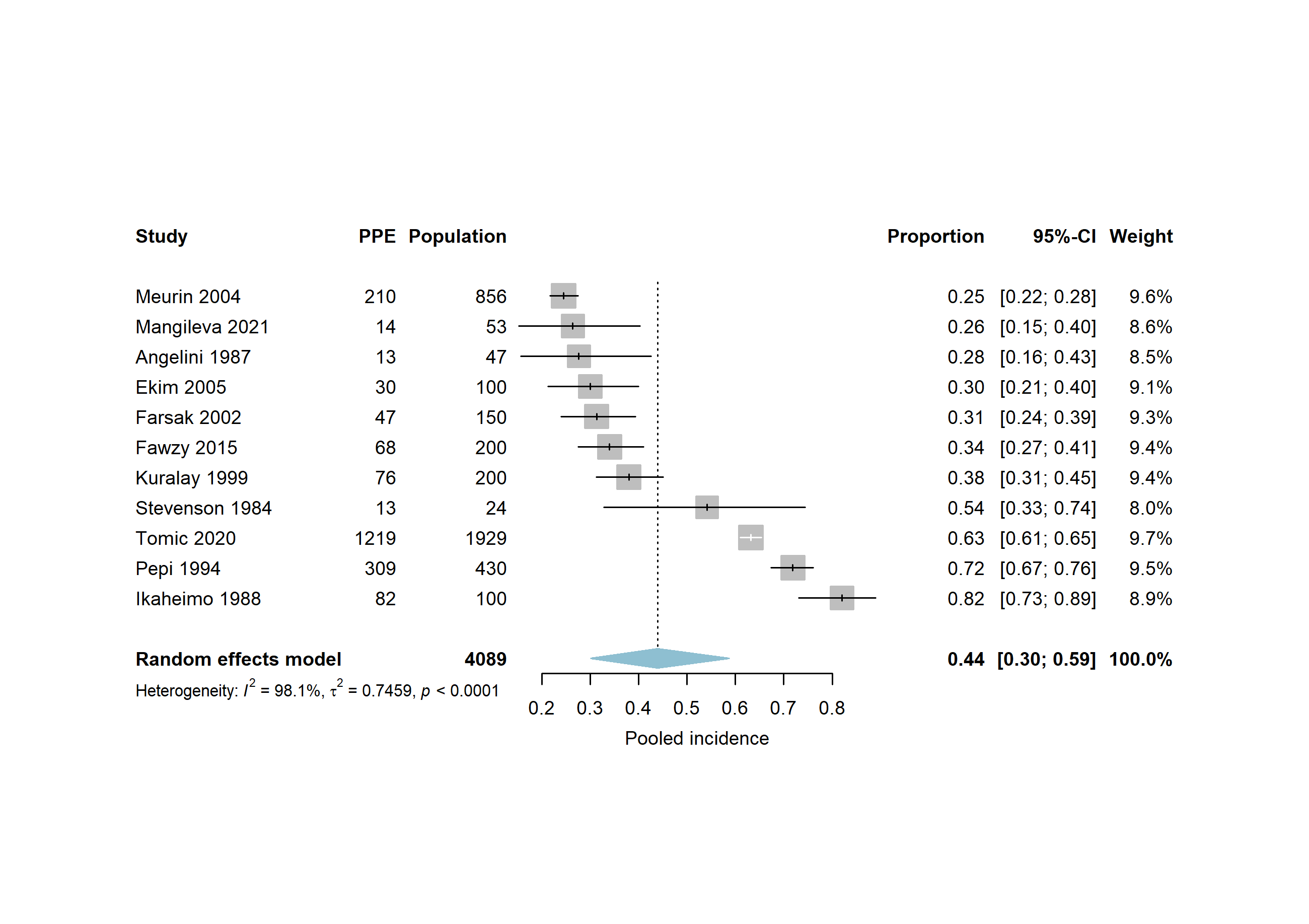


**CABG**

**Valve +/-CABG**

**A**

**B**

**C**

**Supplementary figure 2B.** Plots with random effect meta-analyses providing pooled incidence of PPE per surgery with 95% confidence intervals. (corresponding plots of A: CABG, B: Valve(s)+/-CABG, C: Aorta). Two studies (Bunge et al. 2014, Cakalagaoglu et al. 2012) with 100% PPE incidence (both studies had no ‘no PPE’ category; counting 0-10 mm of PPE as small) were excluded from the analysis.

#### **Supplementary figure 2C.** Plots with PPE incidence per effusion size per study, and total estimated incidence


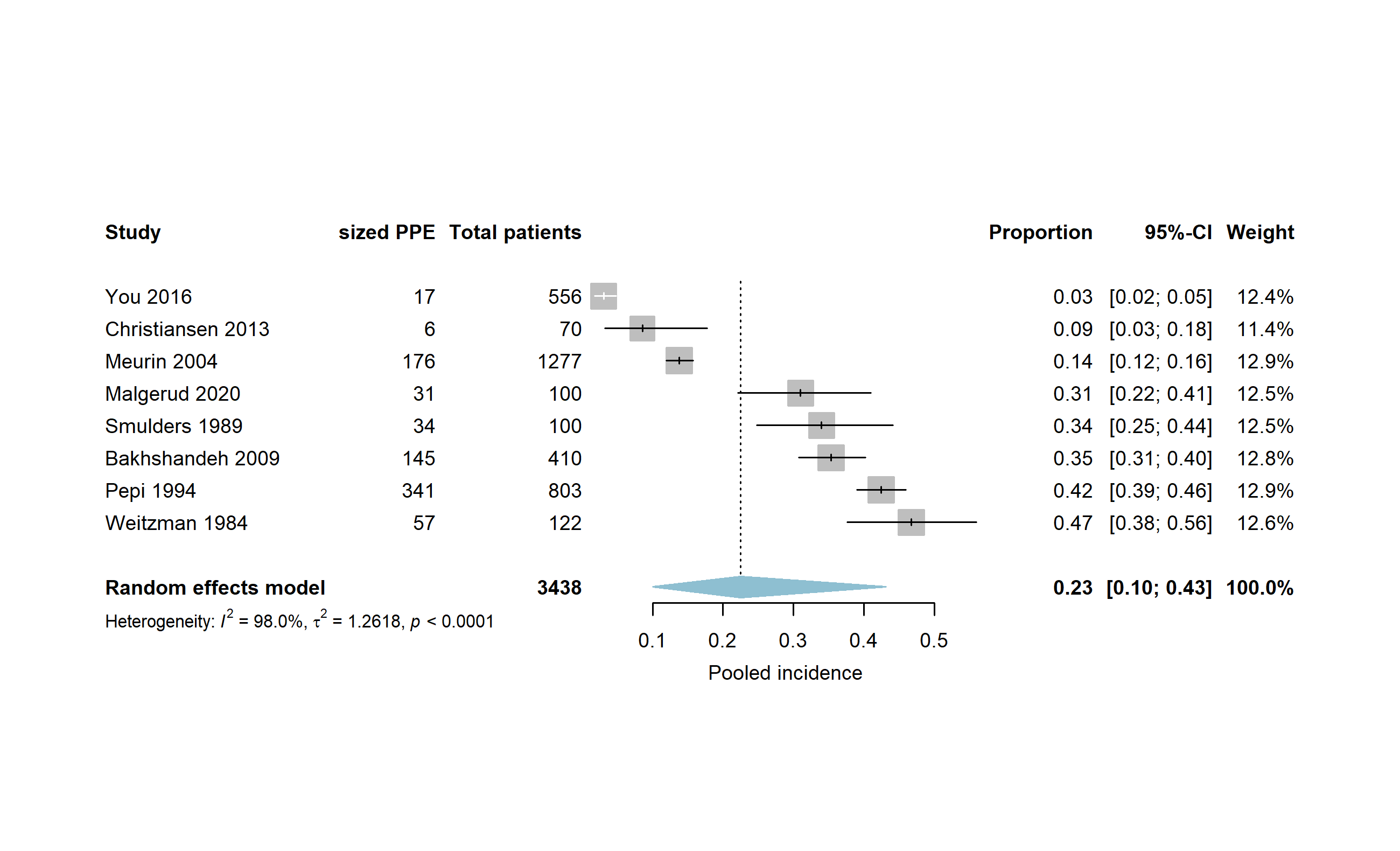

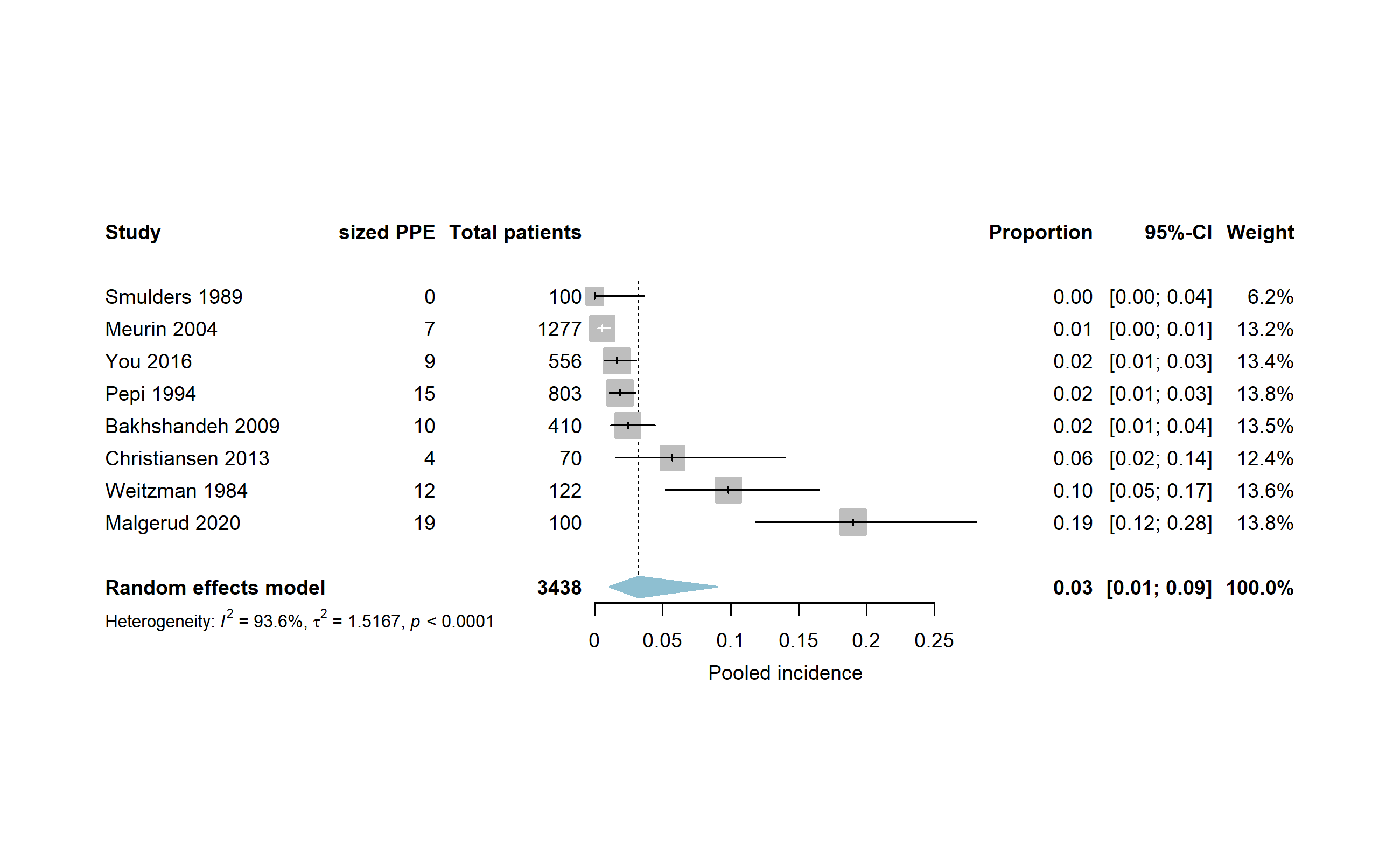

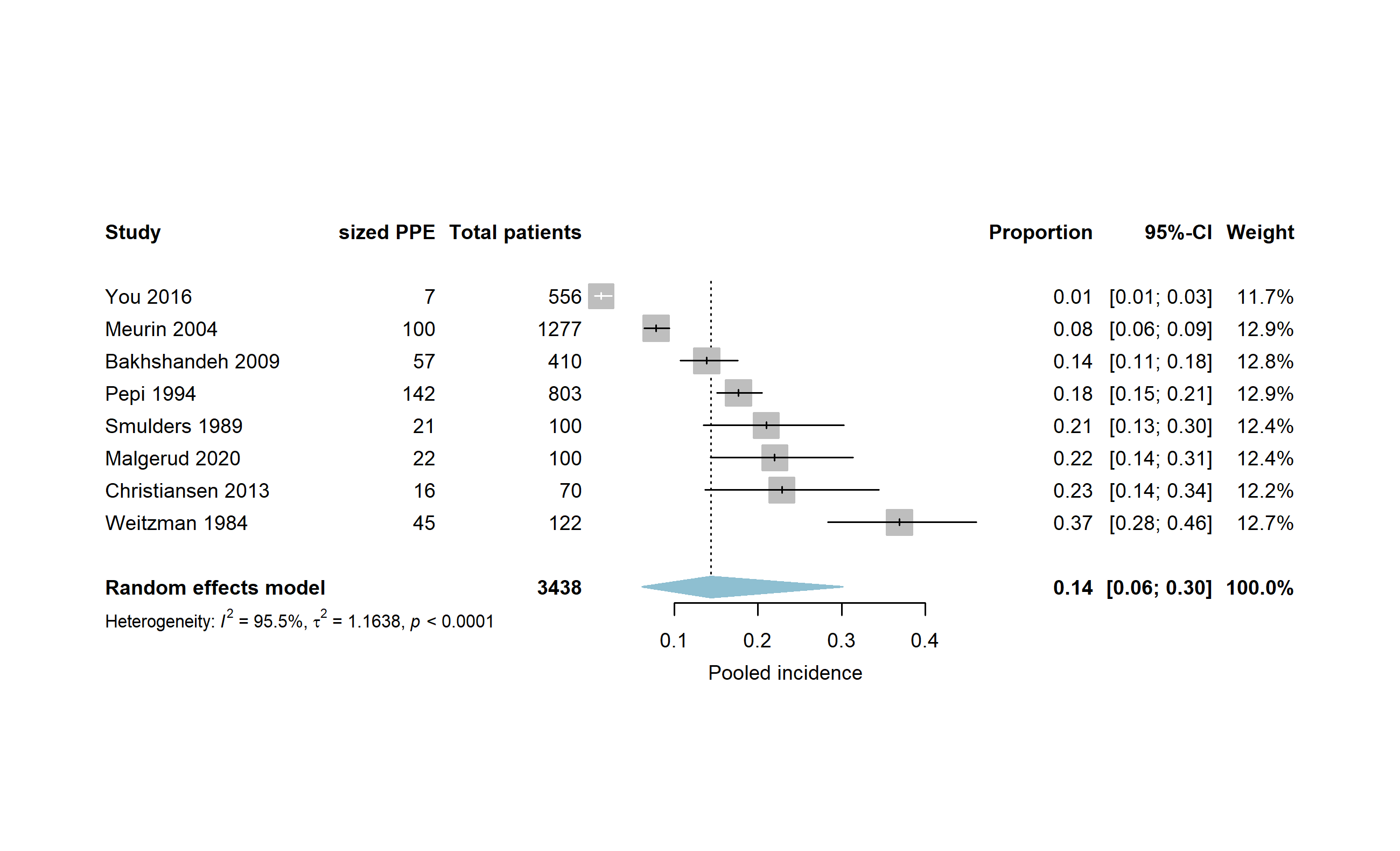


**Large**

**Small**

**Moderate**

**A**

**B**

**C**

**Supplementary figure 2C.** Plots with random effect meta-analyses providing pooled incidence of PPE per effusion size (small, moderate, large) with 95% confidence intervals. (corresponding plots of A: small, B: moderate, C: large). Two studies (Bunge et al. 2014, Cakalagaoglu et al. 2012) with 100% PPE incidence (both studies had no ‘no PPE’ category; counting 0-10 mm of PPE as small) were excluded from the analysis.

**Supplementary figures 3.** Plots with PPE-related reinterventions total estimated incidence, per surgery type and per effusion size

#### **Supplementary figure 3A.** Plot with incidence of PPE-related reinterventions per study and total estimated incidence


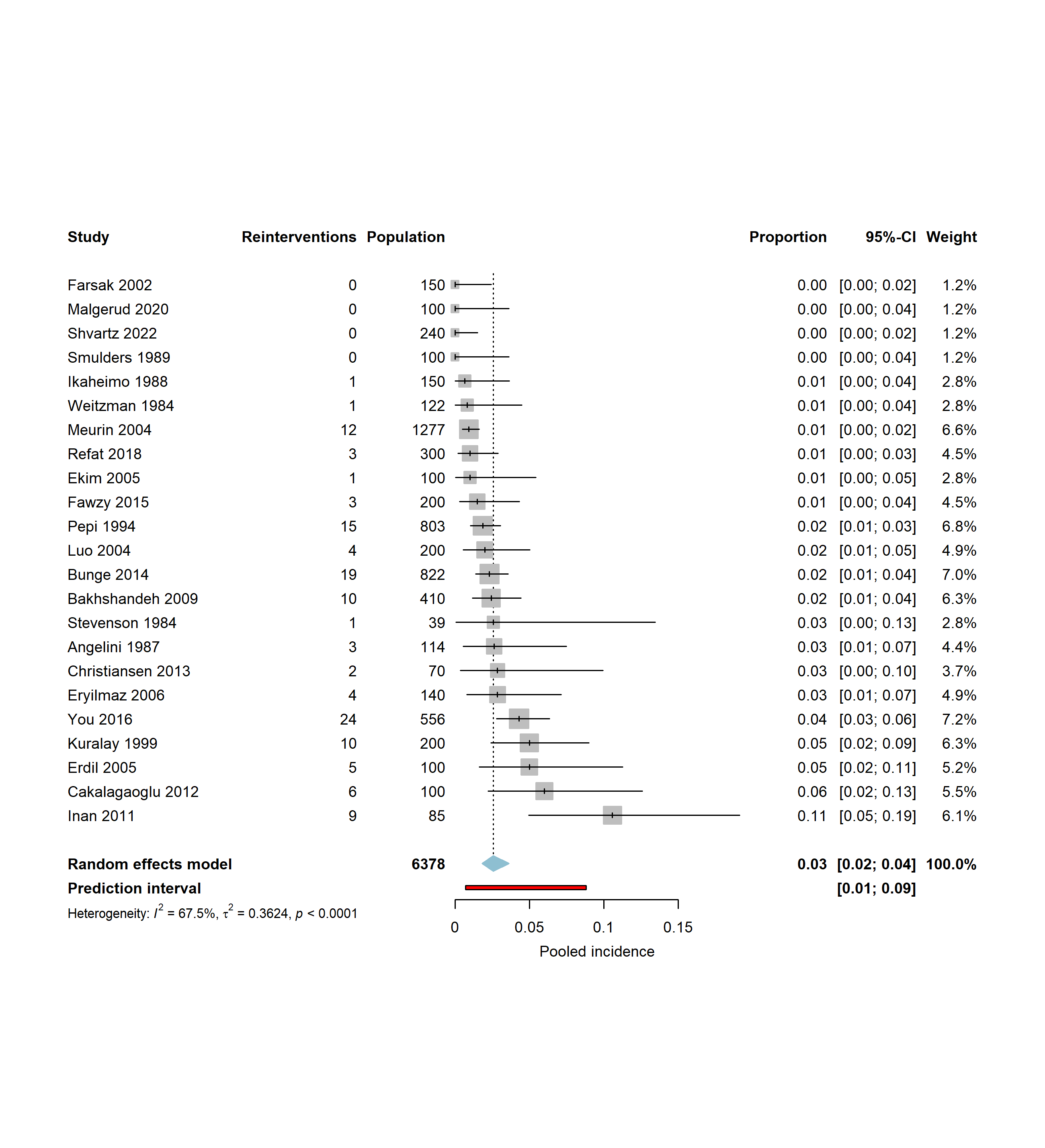


**Supplementary figure 3A.** Plot with random effect meta-analyses providing pooled incidence of PPE-related reinterventions per study with 95% confidence intervals, and total estimated incidence with a prediction interval.

**Supplementary figure 3A.** Plot with random effect meta-analyses providing pooled incidence of routinely assessed PPE-related reintervention per study with 95% confidence intervals, and total estimated incidence. Two studies (Bunge et al. 2014, Cakalagaoglu et al. 2012) with 100% PPE incidence (both studies had no ‘no PPE’ category; counting 0-10 mm of PPE as small) were excluded from the total PPE pooled incidence.

#### **Supplementary figure 3B.** Plots with PPE-related reintervention incidence per type of surgery per study, and total estimated incidence


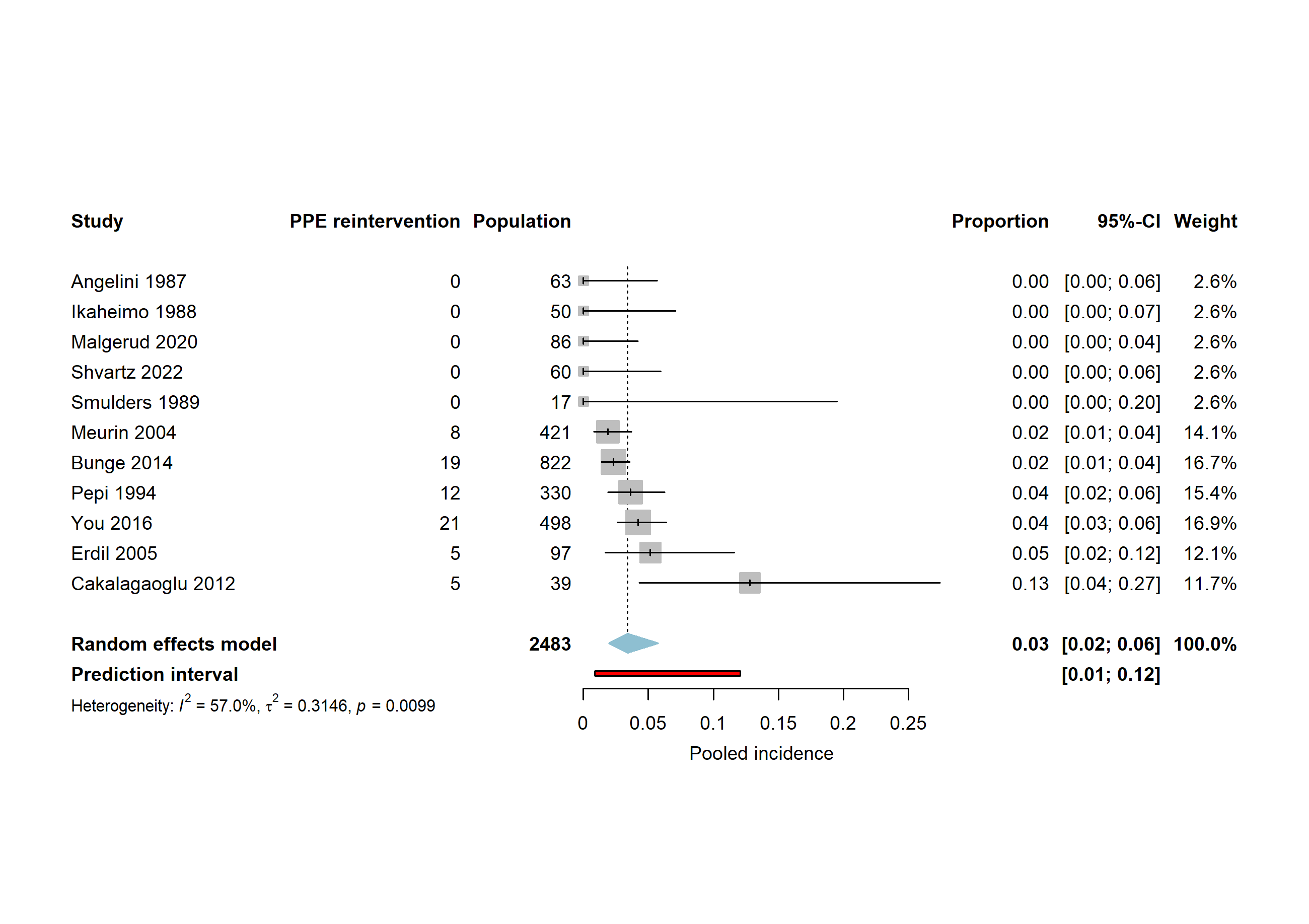

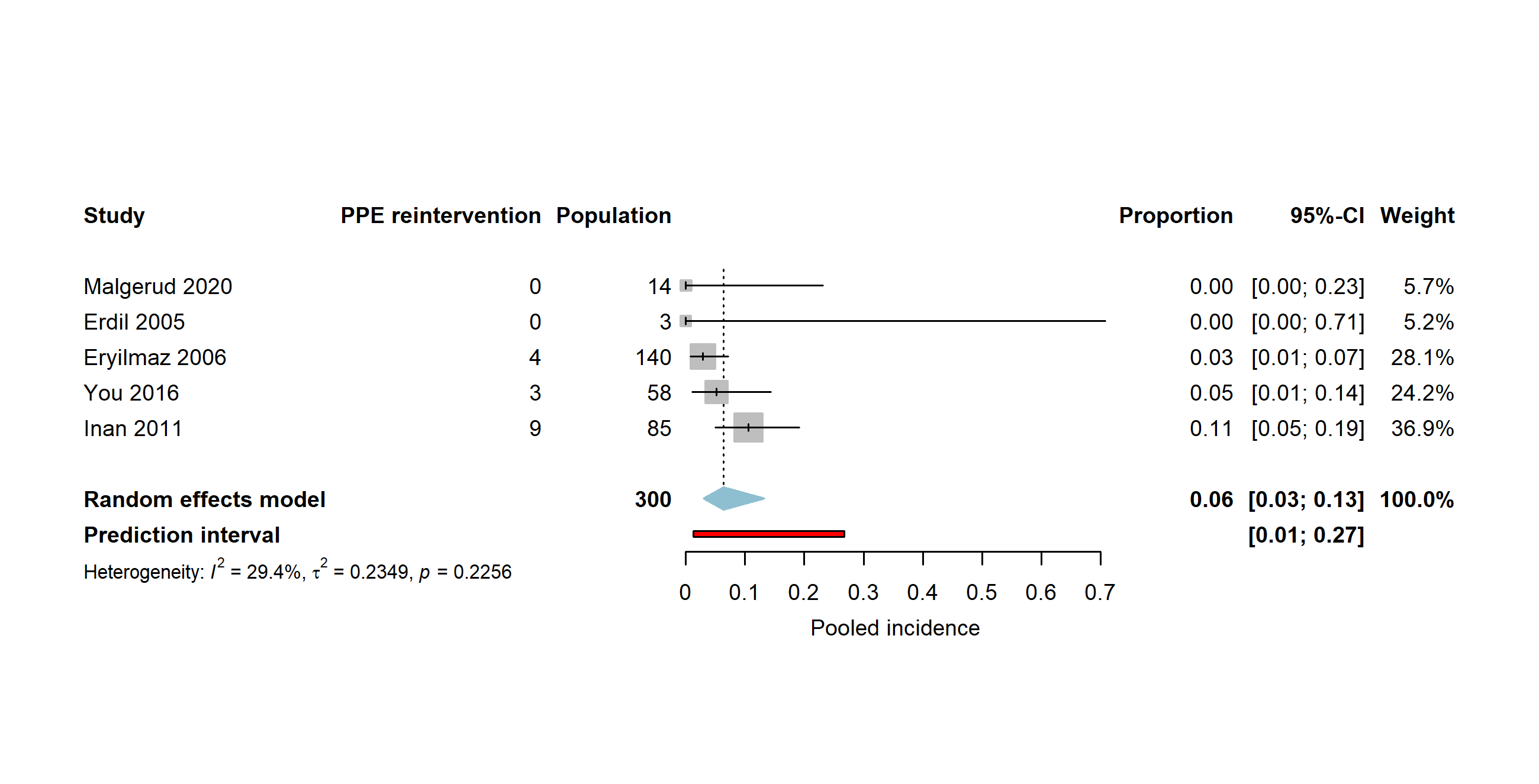

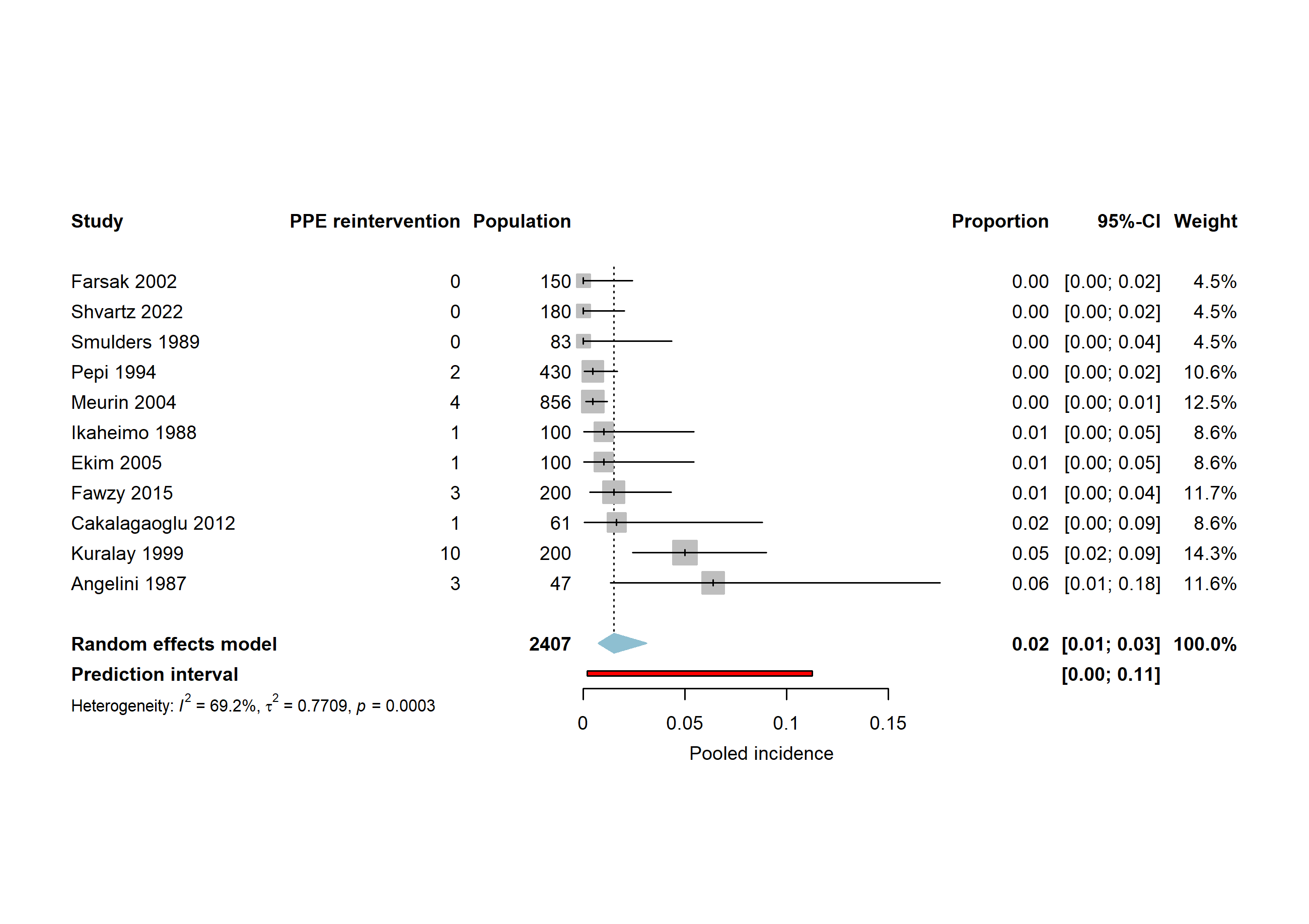


**Aorta**

**CABG**

**Valve +/-CABG**

**A**

**B**

**C**

**Supplementary figure 3B.** Plots with random effect meta-analyses providing pooled incidence of PPE-related reintervention per surgery with 95% confidence intervals, and total estimated incidence with a prediction interval (corresponding plots of A: CABG, B: Valve(s)+/-CABG, C: Aorta).

#### **Supplementary figure 3C.** Plots with PPE-related reintervention incidence per effusion size per study, and total estimated incidence


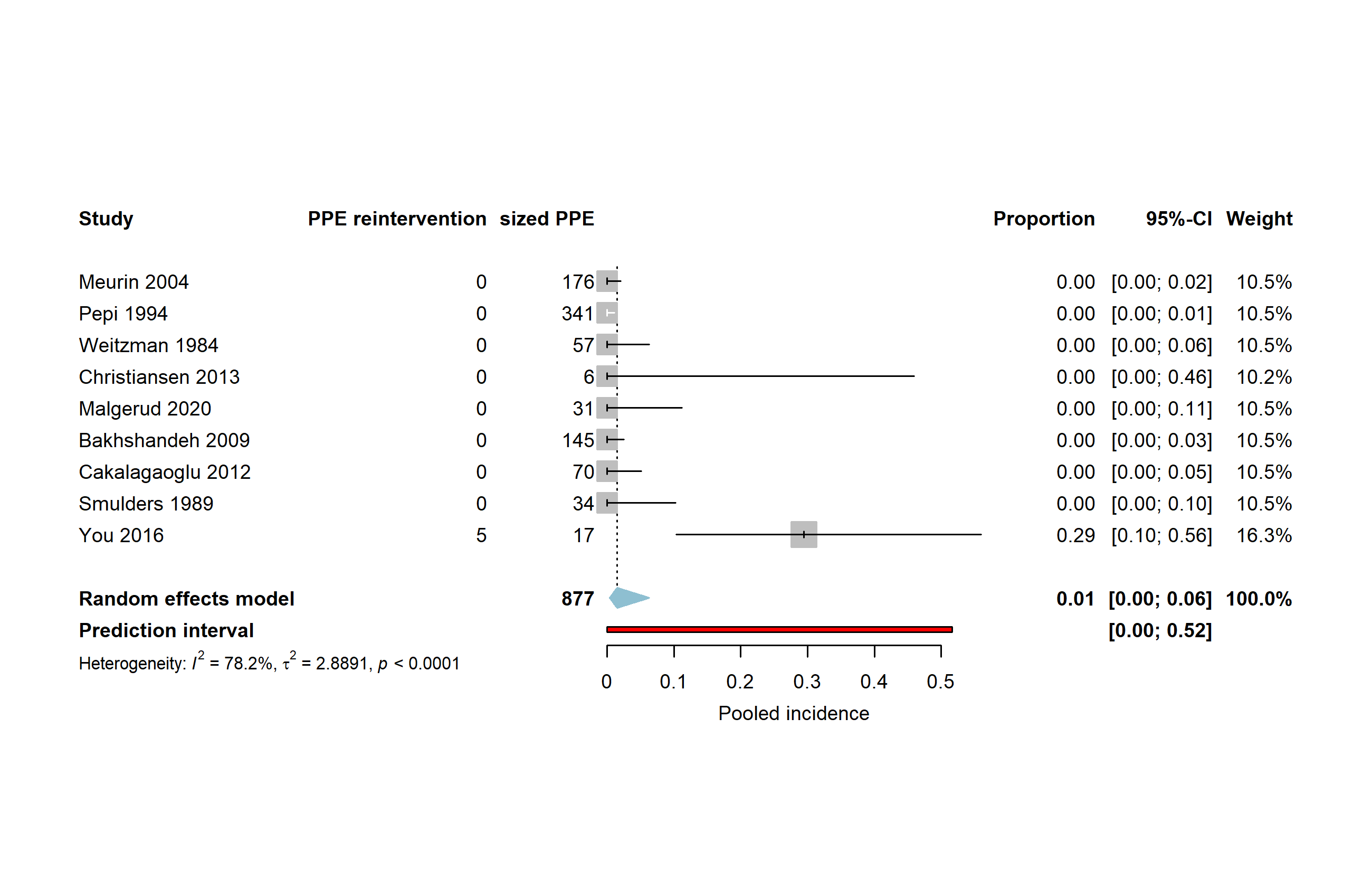

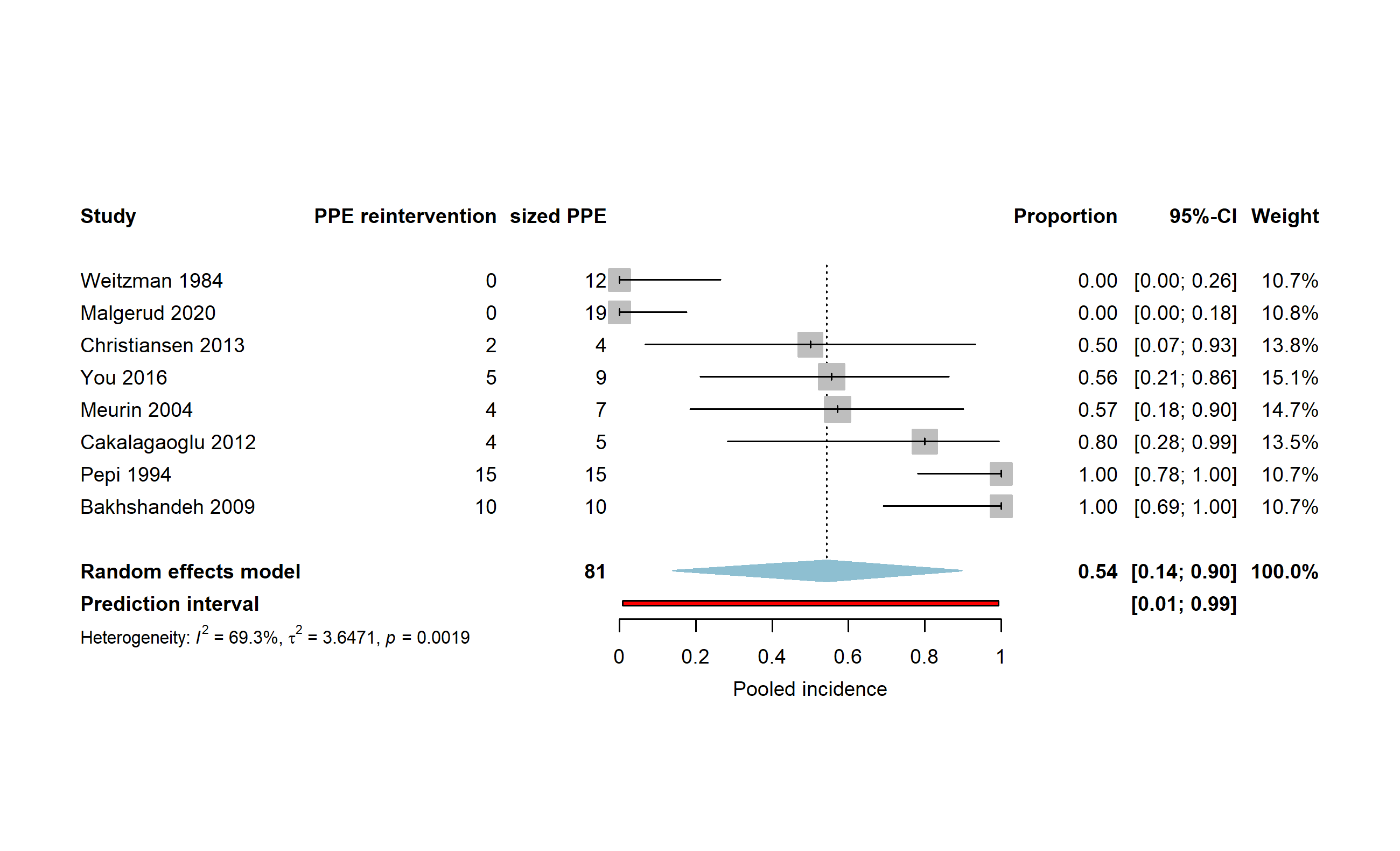

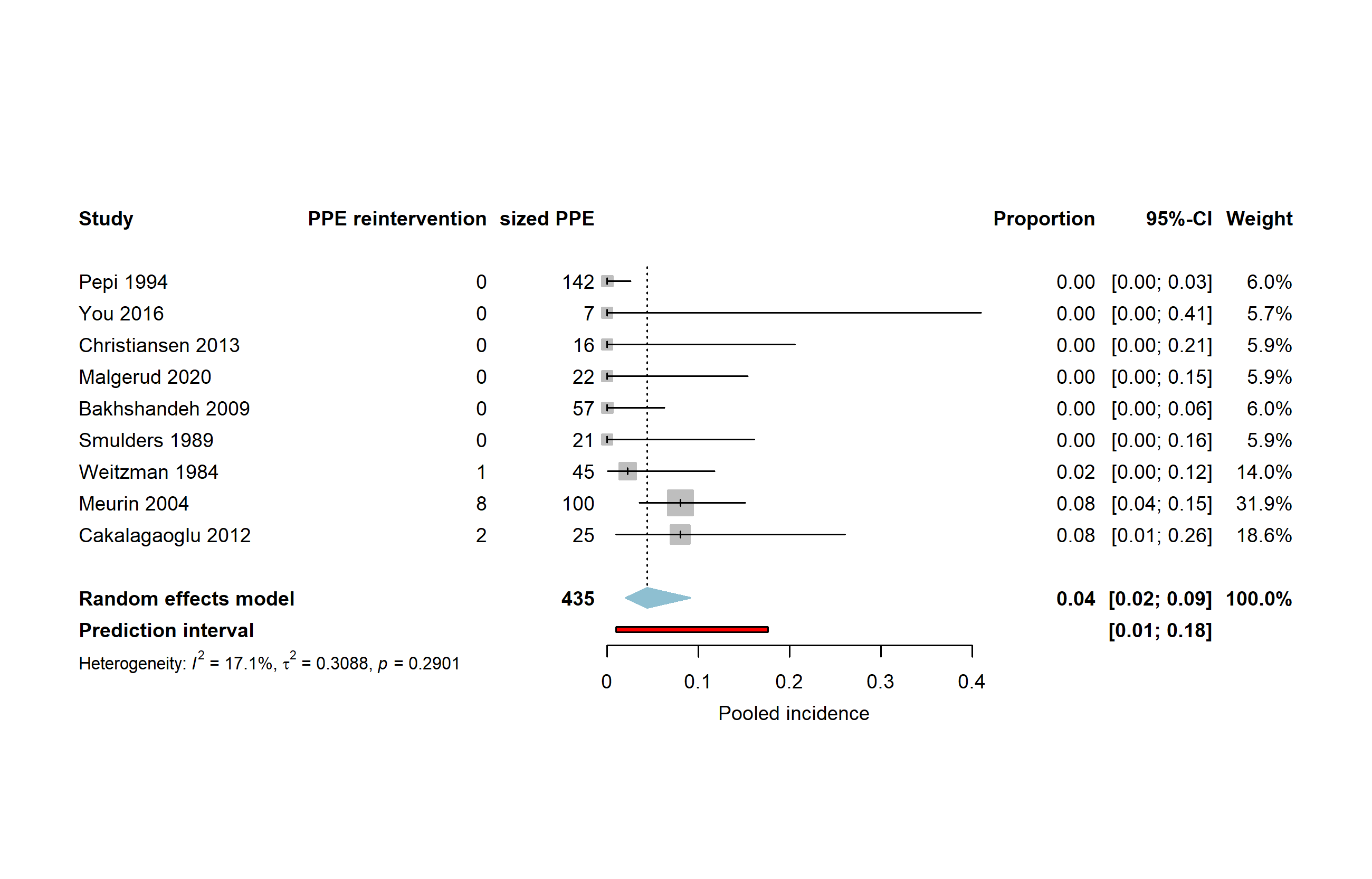


**Large**

**Small**

**Moderate**

**A**

**B**

**C**

**Supplementary figure 3C.** Plots with random effect meta-analyses providing pooled incidence of PPE per effusion size (small, moderate, large) with 95% confidence intervals and total estimated incidence with a prediction interval (corresponding plots of A: small, B: moderate, C: large).

### **Supplementary figure 4**. Reported symptomatology of PPE-related reinterventions


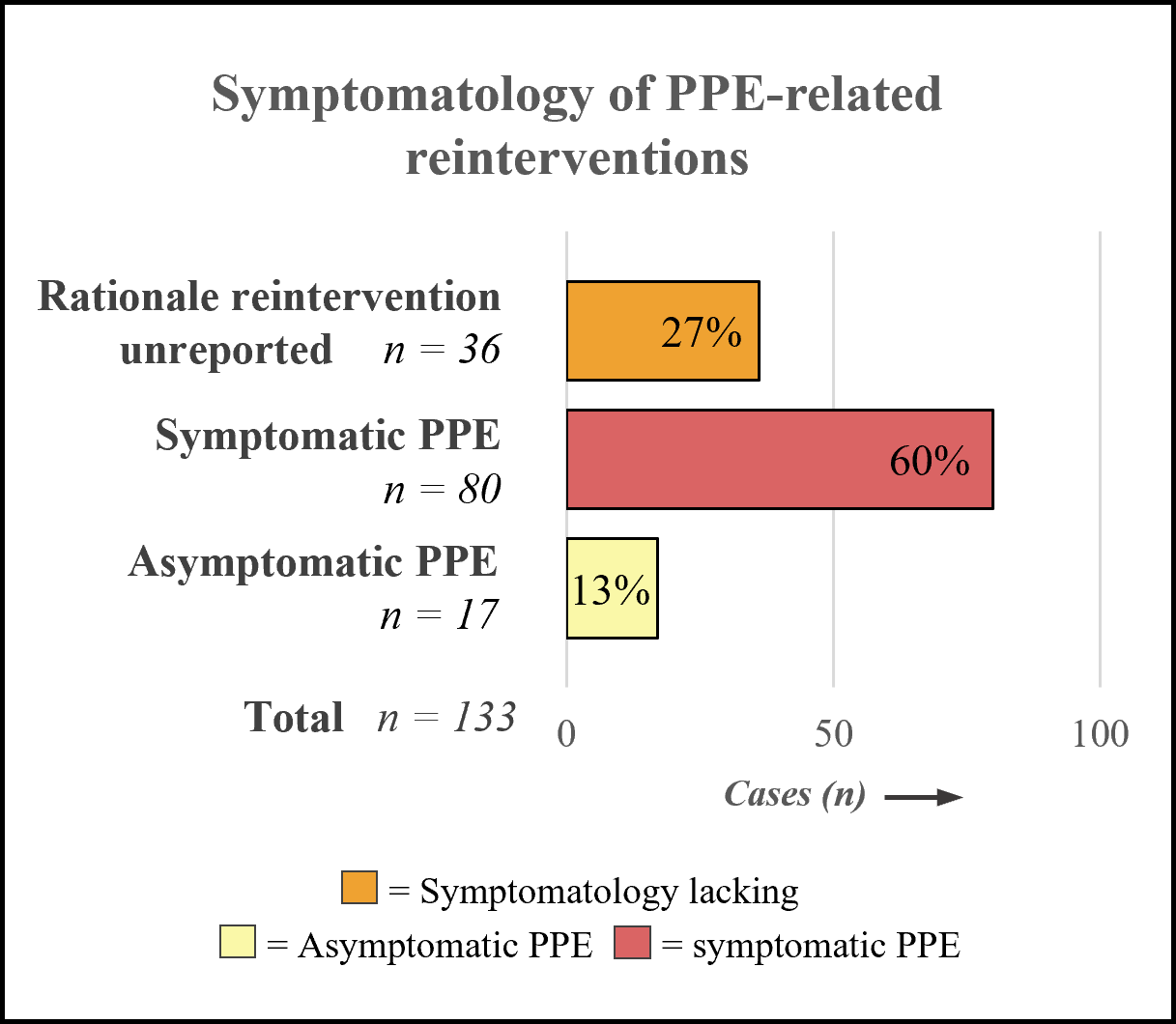


**Supplementary figure 4.** Figure displaying the distribution of the presence or absence of reported symptomatology by the examined studies of patients that had a PPE-related reintervention.

PPE: postoperative pericardial effusion.

**Supplementary tables**

Supplementary table 1 - Search strings Embase, Pubmed, Web of Science

Supplementary table 2a - PECO(S) framework with inclusion and exclusion criteria

Supplementary table 2b - In- and exclusion criteria for full-read analysis

Supplementary table 3 - JBI Checklist Critical Appraisal for studies reporting prevalence data

Supplementary table 4 - Time specification per PPE-related reintervention

Supplementary table 5 - Perioperative interventions affecting the incidence of PPE or PPE-related reinterventions

**Supplementary table 1**. Search strings Embase, PubMed, Web of Science

| **Database** | **Search string** |
| --- | --- |
| PubMed (Medline) | (("Cardiac Tamponade"[Mesh]) OR (Cardiac tamponade*[Title/Abstract])  OR ("Pericardial Effusion"[Mesh]) OR (Pericardial effusion*[Title/Abstract]) OR (Pericardial tamponade*[Title/Abstract]) OR (Hemopericardium[Title/Abstract]))  AND  (("Thoracic Surgery"[Mesh]) OR (Thoracic Surgery[Title/Abstract]) OR  ("Cardiac Surgical Procedures"[Mesh]) OR (Heart Surgery[Title/Abstract]) OR (Cardiac Surgery[Title/Abstract]) OR (Cardiac Surgical Procedure*[Title/Abstract]) OR (Heart Surgical Procedure*[Title/Abstract]) OR (Cardiac Valve Annuloplast*[Title/Abstract]) OR (Heart Valve Annuloplast*[Title/Abstract]) OR (Heart Valve Prosthesis Implantation[Title/Abstract]) OR (Coronary Artery Bypass[Title/Abstract]) OR (Coronary Artery Bypass Surgery[Title/Abstract]))  AND  (("Echocardiography"[Mesh]) OR (Echocardiography[Title/Abstract]) OR (Transthoracic Echocardiography[Title/Abstract]) OR (Transesophageal Echocardiography[Title/Abstract]) OR (Doppler Echocardiography[Title/Abstract]))  AND  ((complicat*[Title/Abstract]) OR (incidence[Title/Abstract]) OR (occur*[Title/Abstract]) OR (follow-up[Title/Abstract]) OR (natural history[Title/Abstract]) OR (management[Title/Abstract]))  NOT (case report*[Title])   - Filter: English Abstracts |
| Embase (Ovid) | (pericardial effusion/ or exp hemopericardium/ or exp heart tamponade/ or "pericardial effusion".ti,ab,kf. or "heart tamponade".ti,ab,kf. or hemopericardium.ti,ab,kf. or "pericardial tamponade".ti,ab,kf. or "cardiac tamponade".ti,ab,kf.)  and  (heart surgery/ or exp coronary artery surgery/ or exp heart valve surgery/ or exp open heart surgery/ or "heart surgery".ti,ab,kf. or "cardiac surgery".ti,ab,kf. or "cardiac surgical procedures".ti,ab,kf. or cardiosurgery.ti,ab,kf. or "heart operation".ti,ab,kf. or "coronary artery surgery".ti,ab,kf. or "coronary artery bypass".ti,ab,kf. or "heart valve surgery".ti,ab,kf. or "open heart surgery".ti,ab,kf.)  and  (exp echocardiography/ or "echocardiograph*".ti,ab,kf. or "transesophageal echocardiograph*".ti,ab,kf. or "transthoracic echocardiograph*".ti,ab,kf. or "cardia* echograph*".ti,ab,kf. or "echocardiogram".ti,ab,kf. or "heart echocardiograph*".ti,ab,kf.)  And  ("complicat*".ti,ab,kf. or "incidence".ti,ab,kf. or “occur*”.ti,ab,kf. or “follow-up” .ti,ab,kf. or “natural history”.ti,ab,kf. or “management”.ti,ab,kf.)  Not case report*.ti.   - Filter: English Abstracts |
| Web of Science (Clarivate) | (TS=(pericardial effusion*) OR TS=(cardiac tamponade*) OR TS=(hemopericardium))  AND  (TS=(cardiac surgery) OR TS=(thoracic surgery) OR TS=(heart surgery) OR TS=(heart surgical procedure*) OR TS=(cardiac surgical procedure*) OR TS=(cardiac valve annuloplast*) OR TS=(heart valve annuloplast*) OR TS=(heart valve prosthesis implantation) OR TS=(heart valve surgery) OR TS=(coronary artery bypass))  AND  (TS=(echocardiograph*) OR TS=(transthoracic echocardiograph*) OR TS=(transesophageal echocardiograph*) OR TS=(doppler echocardiograph*))  AND  (TS=(complicat*) OR TS=(incidence) OR TS=(occur*) OR TS=(follow-up) OR TS=(natural history) OR TS=(management))  NOT TI=(case report*)   - Filter: English |

**Supplementary table 1.** Table with the search strings used for study collection, categorized per database.

**Supplementary table 2a**. PECO(S) framework with inclusion and exclusion criteria

| **PECOS  (see text)** | **Inclusion criteria** | **Exclusion criteria** |
| --- | --- | --- |
| *P*opulation | - All patients after open heart surgical procedures (except congenital or heart transplantation surgery) having received at least 1 routine postoperative echocardiographic examination (TTE or TEE) for pericardial effusion. | - Patients with age <18y old.  - Patients subjected to congenital or heart transplantation surgery.  - Patients with pericardial effusion due to a different etiology than cardiac surgery.  - Animals. |
| *E*xposure | Sub-acute and late cardiac tamponade or significant (symptomatic) pericardial effusion – that being 48 or more hours postoperatively – present on routine postoperative echocardiographic examination (TTE or TEE), requiring either:   - Surgical intervention (re-sternotomy, sub xiphoidal drainage, thoracotomy, surgical drainage through video-assisted thoracoscopy (VATS), etc.)   - Pericardiocentesis | Reintervention due to suspicion of acute bleeding/early cardiac tamponade or pericardial effusion within the first 48 hours after surgery. |
| *C*omparison | - Patients with no pericardial effusion.  - Patients with (asymptomatic) pericardial effusion not requiring intervention, therefore being treated conservatively or being discharged without surgical or percutaneous intervention, or being treated with drug therapy targeting the excessive amount of pericardial effusion. | No suitable comparison group, due to absence of routine postoperative echocardiography (TTE or TEE) in all patients. |
| *O*utcome | At least one of the following items have been measured:  - Incidence of pericardial effusion  - Incidence of cardiac tamponade  Other items of interest (if measured):  - Size of pericardial effusion  - patient characteristics or perioperative factors influencing incidence of pericardial effusion or cardiac tamponade | No incidence of pericardial effusion has been reported (e.g., study reported other echocardiographic features such as ventricular or valvular function, or only reported the average size of pericardial effusion) |
| *S*tudy design | All original research articles, except for case reports.  Studies need to be written in the English language and published in a journal with accessible abstract and with the availability of a full-text read. | Case reports and (systematic) reviews will be excluded but will be analyzed on the original research articles that have been used in writing the review. |

**Supplementary table 2a.** Table with an overview of the in- and exclusion criteria in accordance with the PECO(S) framework, used for building the search strings and article selection

**Supplementary table 2b**. In- and exclusion criteria for full-read analysis

| **Inclusion criteria** | **Exclusion criteria** |
| --- | --- |
| - Adult patients (aged ≥18 years) after open heart surgery - At least one postoperative echocardiogram reporting the presence or absence of PPE was conducted ≥48 hours post-surgery. - The postoperative echocardiogram was performed on a routine basis in the whole study cohort, regardless of symptomatology. | - Congenital surgery, heart transplantation or emergency surgery after traumatic injury. - Echocardiography data was only available for patients with symptomatic course. - Non-English language or lack of full-text availability. |

**Supplementary table 2b.** Table with a summarized overview of the in- and exclusion criteria used for inclusion of studies during the full-read analysis phase.

**Supplementary table 3.** JBI Checklist Critical Appraisal for studies reporting prevalence data

(for whole checklist, see: [JBI_Critical_Appraisal-Checklist_for_Prevalence_Studies2017_0.pdf](https://jbi.global/sites/default/files/2019-05/JBI_Critical_Appraisal-Checklist_for_Prevalence_Studies2017_0.pdf))

| **Study** | **1. Appropriate sample for target population** | **2.  Adequate patient sample selection** | **3.  Adequate patient sample size (power analysis)** | **4.  Adequate description of study subject and setting** | **5.  sufficient coverage of sample subgroups** | **6.  Valid methods used for diagnosis of condition** | **7.  Studied condition measured reliably for all participants** | **8. Valid statistical analysis** | **9.  Valid data availability, drop-outs adequately managed** | **Scored points/ total points** | **Further explanation or reason for exclusion (if more than 2 ‘X’)** |
| --- | --- | --- | --- | --- | --- | --- | --- | --- | --- | --- | --- |
| Alsaddique 2015 | ***✓*** | *X* | n.a. | **✓** | n.a. | **✓** | **✓** | **✓** | *X* | 5*/*7 | *- Of 275 patients included, only 91 were recruited.*  *- No clearly described drop-out management* |
| Angelini 1987 | ***✓*** | ***✓*** | n.a. | **✓** | n.a. | **✓** | **✓** | **✓** | **✓** | 7*/*7 |  |
| Bakhshandeh 2009 | **✓** | **✓** | *X* | **✓** | **✓** | **✓** | **✓** | **✓** | **✓** | *8/9* | *- No sample size power analysis.* |
| Bunge 2014 | **✓** | **✓** | **✓***/X* | **✓** | **✓** | **✓** | **✓** | **✓** | **✓** | *8*-9*/9* | *- No sample size power analysis (yet large patient population  (n = 822)).* |
| Cakalagaoglu 2012 | **X** | **✓** | *X* | **✓** | **✓** | **✓** | **✓** | **✓** | **✓** | 7*/9* | *- Broad set of exclusion criteria.*  *- No sample size power analysis.* |
| Christiansen 2013 | **✓** | **✓** | n.a. | **✓** | **n.a.** | **✓** | **✓** | **✓** | **✓** | *7/*7 |  |
| Ekim 2005 | *X* | **✓** | *X* | **✓** | **✓** | **✓** | **✓** | **✓** | **✓** | 7*/*9 | *- Broad set of exclusion criteria.*  *- No sample size power analysis.* |
| Erdil 2005 | **✓** | **✓** | *X* | **✓** | **✓** | **✓** | **✓** | **✓** | **✓** | *8/9* | *- No sample size power analysis.* |
| Eryilmaz 2006 | **✓** | **✓** | *X* | **✓** | **✓** | **✓** | **✓** | **✓** | **✓** | *8/9* | *- No sample size power analysis.* |
| Farsak 2002 | *X* | **✓** | *X* | **✓** | **✓** | **✓** | **✓** | **✓** | **✓** | *7/9* | *- Broad set of exclusion criteria.*  *- No sample size power analysis.* |
| Fawzy 2015 | *X* | **✓** | *X* | **✓** | **✓** | **✓***/* *X* | **✓** | **✓** | **✓** | *6*-7*/9* | *- Broad set of exclusion criteria.*  *- No sample size power analysis.*  *- Study reports various PPE sizes, yet not described in methods*  *🡪 included due to relevance and credibility of data, as well as big population size (n = 200)* |
| Ikaheimo 1988 | **✓** | *X* | *X* | **✓** | **✓** | **✓** | **✓** | **✓** | *X* | *6/9* | *- Not clearly described if group 2 and 3 were randomized.*  *- No sample size power analysis.*  *- according to table III 12/150 patients had no 2w follow-up, yet no drop-outs described*  *🡪 included due to relevance of observational results of the study (lack of patient selection description less relevant for the main research question)* |
| Inan 2011 | **✓** | **✓** | *X* | **✓** | **✓** | **✓** | *X* | **✓** | **✓** | *7/9* | *- No sample size power analysis.*  *- Only PPE requiring intervention is reported (yet routine TTE examination of PPE is reported)* |
| Kuralay 1999 | *X* | **✓** | *X* | **✓** | **✓** | **✓** | **✓** | **✓** | **✓** | *7/9* | *- Broad set of exclusion criteria.*  *- No sample size power analysis.* |
| Luo 2004 | **✓** | **✓** | n.a. | **✓** | n.a. | **✓** | **✓** | **✓** | **✓** | 7/7 |  |
| Malgerud 2020 | **✓** | **✓** | **✓** | **✓** | **✓** | **✓** | **✓** | **✓** | **✓** | 9/9 |  |
| Mangileva  2021 | **✓** | **✓** | X | **✓** | **✓** | **✓** | **✓** | **✓** | **✓** | 8/9 | - No sample size power analysis. |
| Meurin 2004 | **✓** | **✓** | n.a. | **✓** | n.a. | **✓** | **✓** | **✓** | **✓** | 7/7 |  |
| Pepi 1994 | **✓** | **✓** | n.a. | **✓** | n.a. | **✓** | **✓** | **✓** | **✓/X** | 6-7/7 | - Concerning point 9: quite some loss of follow-up on POD 14 and 25, but this is substantiated by the choice to discharge patients from follow-up with small PPE. |
| Refat 2018 | **✓** | **✓** | **✓** | **✓** | **✓** | **✓** | **✓** | **✓** | X | 8/9 | - Drop-outs/loss of follow-up (n = 7/300) after 3 months not described |
| Shvartz 2022 | **✓** | **✓** | **✓** | **✓** | **✓** | **✓** | **✓/** X | **✓** | **✓** | 8-9/9 | - Concerning PPE-related reinterventions: table 3 reports 0 reinterventions, however the percentual incidence indicates 1 reintervention |
| Smulders 1989 | **✓** | **✓** | X | **✓** | **✓** | **✓** | **✓** | **✓** | **✓** | 8/9 | - No sample size power analysis. |
| Stevenson 1984 | **✓** | X | n.a. | **✓** | n.a. | **✓** | **✓** | **✓** | **✓** | 6/7 | - No clear explanation of inclusion process (also all patients included by 1 person) |
| Tomic 2020 | **✓** | **✓** | X | **✓** | **✓** | **✓** | **✓** | **✓** | **✓** | 8/9 | - No sample size power analysis. |
| Weitzman 1984 | **✓** | **✓** | n.a. | **✓** | n.a. | **✓** | **✓** | n.a. | **✓** | 6/6 | (No statistical differences tested) |
| You 2016 | **✓** | **✓** | n.a. | **✓** | n.a. | **✓** | **✓** | **✓** | **✓** | 7/7 |  |

**Supplementary table 3.** Table with the critical appraisal scores using the JBI Checklist for studies reporting prevalence data, alongside with description in case of lacking points. The maximum number of points was reduced if no intervention was studied.

**Supplementary table 4**. Time specification per PPE-related reintervention

**Supplementary table 4.** Table with the total number of reinterventions from the 19/26 studies with available data on timing of reintervention. Reinterventions are categorized per surgery type, before POD 30 and after POD 30.

AVR: aortic valve replacement; CABG: coronary artery bypass grafting; POD: postoperative day.

#Pulmonary embolectomy.

| **PPE-related reintervention** | **N** | **Day of incidence (n, %)** | |
| --- | --- | --- | --- |
|  |  | **POD <30** | **POD ≥30** |
| Total reinterventions with time specification | 83 | 49 (59) | 34 (41) |
| (isolated) CABG  Valve(s)±CABG  Aorta  Other**^#^** | 22  51  9  1 | 7 (32)  36 (71)  5 (56)  1 (100) | 15 (68)  15 (29)  4 (44)  0 |

**Supplementary table 5**. Perioperative interventions affecting the incidence of PPE or PPE-related reinterventions

| **Intervention** | **Study type** | **Study** | **Study size (n)** | | **Incidence PPE  (n, %)** | | **Incidence reintervention (n, %)** | | **Statistical sign.** |
| --- | --- | --- | --- | --- | --- | --- | --- | --- | --- |
|  |  |  | **INV** | **CON** | **INV** | **CON** | **INV** | **CON** |  |
| **Intraoperative interventions** |  |  |  | |  | |  | |  |
| *Posterior pericardiotomy (PPT) (one drain in the anterior mediastinum, one in left pleural cavity vs. control, single drain in anterior mediastinum)* | Prosp. RCT  Prosp. RCT  Prosp. RCT  Prosp. RCT  Prosp. RCT  Multicenter Prosp. RCT  Prosp. RCT | Bakhshandeh 2009  Cakalagaoglu 2012  Ekim 2005  Erdil 2005  Farsak 2002  Fawzy 2015  Kuralay 1999 | 198 (PPT)  50 (PPT)  50 (PPT)  50 (PPT)  75 (PPT)  100 (PPT)  100 (PPT) | 194  50  50  50  75  100  100 | 20 (10)  0  6  4  8 (11)*  0**^†^**  15 (15)  0 | 194 (100)**^†^**  30**^^^**   21  19  32 (39)*  7 (9)**^#^**  50 (50)  21 (21) | 0  0  0  0  0  0  0 | 10 (5)  6 (12)  1  5  0  3  10 (10) | **< .05**  PPE **<.001** reint **<.05**  PPE **<.001**  PPE **<.003**  reint .056  **<.0001***  **<.013^#^**  PPE **<.001**  reint .246  PPE **<.001**  reint **.001** |
| *CABG with arterial (LIMA) graft vs. venous graft on LAD* | Retrosp. Case-control | Tomic 2020 | 1468 (LIMA) | 461 | 931 (63) | 288 (62) | - | - | n.s. |
| *Single dose dexamethasone (1.0mg/kg, max dose 100 mg) intravenously after induction of anesthesia vs. placebo* | Double-blinded RCT | Bunge 2014 | 421 (Dexa) | 401 | 22 (5) | 14 (3) | 11 (3) | 8 (2) | n.s. |
| **Chest tube management** |  |  |  |  |  |  |  |  |  |
| *Two chest tubes (one in anterior and one in posterior mediastinum) vs. a single chest tube in anterior mediastinum* | Prosp. Observational cohort | Angelini 1987 | 59 (2 tubes) | 55 | 6 (10) | 29 (53) | 0 | 3 (5) | PPE **<.001** |
| *One chest tube retrosternally and one retrocardiac tube vs. two tubes retrosternally.* | Prosp. RCT | Refat 2018 | 150 (with retrocardiac) | 150 | 1 (1) | 14 (10) | 0 | 3 (2) | PPE **.001** reint **.013** |
| *One active clearance chest tube (PleuraFlow® with FlowGlide^TM^) and standard chest tube vs. 2 standard chest tubes* | Prosp. RCT | Malgerud 2020 | 50 (PleuraFlow) | 50 | 38 (76) | 34 (68) | 0 | 0 | PPE 0.50 |
| *Chest tube length of stay of 24 hours vs. 48 hours postoperatively* | Prosp. RCT | Smulders 1989 | 50 (24h) | 50 | 26 (52) | 29 (58) | 0 | 0 | PPE >0.3 |
| *One large (32F) chest tube in anterior mediastinum and one thin (16F) closed-suction (redon) retrocardiac chest tube vs. single 32F chest tube in anterior mediastinum* | Prosp. RCT | Eryilmaz 2006 | 70 (32F+16F) | 70 | 0 | 6 (9) | 0 | 4 (6) | PPE **.001**  reint .120 |
| **Postoperative interventions** |  |  |  |  |  |  |  |  |  |
| *Postoperative (+ 2 weeks before surgery) administration of aspirin 250 mg/day + dipyridamole 225 mg/day vs. warfarin (VKA) therapy* | Prosp. cohort | Ikaheimo 1988 | 50 (VKA) | 50 | 42 (84) | 40 (80) | 0 | 1 (2) | n.s. |
| *Postoperative (+ 7 days before surgery) administration of indomethacin 75 mg/day for 6 weeks vs. placebo* | Prosp. RCT | Inan 2011 | 41 (Indo) | 44 | 1 | 8 | 1 | 6 | PPE **.019**  reint .066 |
| *Postoperative administration of ibuprofen (NSAID) 1200 mg/day in patients with PPS vs. prednisolone 0.5 mg/kg/day in patients with PPS with no improvement on ibuprofen therapy****^‡^*** | Prosp. non-controlled | Mangileva 2021 | 32 (NSAID effective) | 21 | 16 (50) | 18 (86) | - | - | PPE **.008** |
| *Postoperative (+ 24 hours before surgery) administration of colchicine 1 mg/day on day 2-5 after surgery vs. placebo* | Prosp. double-blinded RCT | Shvartz 2022 | 113 (Colchicine) | 127 | 11 (10) | 20 (16) | 0 | 0 | PPE .146 |

**Supplementary table 5.** Overview of included studies describing perioperative interventions or prophylactic administration of medication, and their corresponding effect on the incidence of pericardial effusion and/or PPE-related reintervention.

CON: conservative group; INV: interventional group; PPE: postoperative pericardial effusion; Reint: reintervention group.

*early incidence (within POD 30); ^#^late incidence (from POD 30); ^†^Study describes 20/205 patients with only small pericardial effusion in the intervention group versus 157 small, 30 moderate, 5 large and 2 very large effusion in control group; ^Study scored every patient as small PPE or higher, so only moderate to very large PPE was counted towards incidence; *^‡^*Progression of PPS noted as persisting/progressing pleural or pericardial effusion, positive trends of CRP or ESR, or persisting body temperature >37.0c.
