# Supplementary material for: Postoperative pericardial effusion on routine echocardiography: A review of incidence, progression, and management: To dissolve or to be resolved
Source: Neth Heart J. 2026 Jun 5;34(7-8):254–64. doi: 10.1007/s12471-026-02054-6 (PMC13375986; doi:10.1007/s12471-026-02054-6)
Supplement: Supplementary file 2 — ESM2: Supplementary material 2 [file 12471_2026_2054_MOESM2_ESM.docx]

# **Tables**

## **Table S1.** Overview included studies and corresponding characteristics

| **Author, year, journal** | **Type of study** | **No. of pat (n)** | **Surgery type (n)** | **Intervention for PPE reduction described** | **Moments of routine TTE** | **Different PPE sizes used in study (mm)** | | | | | **Incidence of PPE (n, % of population)** | **Incidence of reintervention (n, % of PPE size,**  **% of total PPE)** | **Timing and incidence of PPE reintervention (n)** | | | | |
| --- | --- | --- | --- | --- | --- | --- | --- | --- | --- | --- | --- | --- | --- | --- | --- | --- | --- |
|  |  |  |  |  |  | **Size** | **Cir** | | | **Loc** |  |  |  |  |  |  |  |
| Alsaddique 2015,  *Heart Lung Circ [51]****^✝^*** | Prospective cohort | 91 | CABG (54)  Valve(s) (33)  CABG + valve (11)  Aorta (1)  LV aneurysm (1) | No | 1. POD 1 2. After removal chest tubes 3. Before discharge 4. 1 mo after discharge | No PPE sizes described | | | | | *Total:* 11 (12.1) | No reintervention incidence described | N.a. | | | | |
| Angelini 1987,  *Eur J Cardiothorac Surg [36]* | Prospective observational cohort | 114 | CABG (47)  Valve(s) (52)  CABG + valve (11)  Septal defects (2)  LV aneurysm (2) | Retrocardiac drainage (vs single anterior tube) | 1. Between POD 3 and 5 | S  M  L | <5  5-15  ≥15 | | | | 14  17  4  *Total:* 35 (30.7) | 0  M + L combined:  3 (14.2)  *Total*: 3 (8.6) | POD 5-18 | | 3 | | |
| Bakhshandeh 2009,  *Asian Cardiovasc Thorac Ann [52]* | Prospective randomized case-controlled | 410 | CABG (274)  Valve(s) (83)  CABG + Valve (53) | Posterior pericardiotomy | 1. Before discharge 2. POD 15 3. POD 30 | S  M  L  VL | <10  10-20  >20  >20 with compression of heart | | | | 145 (35.4)  57 (13.9)  8 (2.0)  4 (1.0)  *Total:* 212 (51.7) | 0  0  L + VL combined:  10 (83.3)  *Total:* 10 (4.7) | No timing described | | | | |
| Bunge 2014,  *Am Heart J [16]* | Randomized, double-blind, placebo controlled trial | 832 | Valve(s) (565)  CABG + Valve (257) | At anesthesia induction i.v. dexamethasone dose | 1. POD 5 | S  M  L | <10  10-20  >20 | | | | 786 (95.6)  26 (3.2)  10 (1.2)  *Total:* 832 (100)* | -  -  -  *Total: 19 (2.3)* | No timing described | | | | |
| Cakalagaoglu 2012,  *Heart Surg Forum [53]* | Prospective, randomized case-controlled | 1 | CABG (61)  Valve(s) (23)  CABG + valve (16) | Posterior pericardiotomy | 1. POD 1 2. before discharge 3. POD 15 4. POD 30 | S  M  L | <10  10-20  >20 | | | | 70 (70.0)  25 (25.0)  5 (5.0)  *Total:* 100 (100)* | 0  2 (8.0)  4 (80.0)  *Total:* 6 (6.0) | POD 7 and 10  (predischarge)  Post-discharge | | | 2  4 |  |
| Christiansen 2013,  *Scand Cardiovasc J [20]* | Prospective observational | 70 | CABG (35)  Valve(s) (25)  CABG + valve (10) | No | 1. POD 4 2. POD 30 | S  M1  M2  L | <10  10-14  >14 | | | <10  10-14  15-19  >19 | 6 (8.6)  10 (14.3)  6 (8.6)  4 (5.7)  *Total:* 26 (37.1) | 0  0  0  2 (50.0)  *Total:* 2 (7.7) | POD 4_  POD 30 | 1  1 | | | |
| Ekim 2005,  *Med Sci Monit [54]* | Prospective randomized | 100 | CABG (100) | Posterior pericardiotomy | 1. POD 3 2. POD 5 3. Before discharge 4. POD >30 (first control) | No PPE sizes described | | | | | *Total:* 30 (30.0) | *Total:* 1 (3.3) | POD <30  POD >30 | | 0  1 | | |
| Erdil 2005,  *J Card Surg [55]* | Prospective consecutive randomized | 100 | Valve(s) (100; MVR (55), AVR (23), AVR + MVR (19), Bentall (3) (all mechanical)) | Posterior pericardiotomy | 1. POD 3 2. Before discharge 3. 1 mo after discharge | No PPE sizes described | | | | | *Total:* 32 (32.0) | *Total:* 5 (15.6) | POD <30  POD >30 | | 0  5 | | |
| Eryilmaz 2006,  *J Thorac Cardiovasc Surg [37]* | Prospective randomized | 140 | Aorta (140; AAR (30), Bentall (98), VSRR (12)) | Retrocardiac drainage (vs single anterior tube) | 1. POD 1 2. POD 7 3. On indication if PPE was present at POD 7 | No PPE sizes described | | | | | *Total:* 16 (11.4) | *Total:* 4 (25.0) | POD <7  POD >7 | | 0  4 | | |
| Farsak 2002,  *Eur J Cardiothorac Surg [56]* | Prospective randomized | 150 | CABG (150) | Posterior pericardiotomy | 1. POD 1 2. POD 5 3. Before discharge 4. 1 mo after discharge | No PPE sizes described | | | | | *Total:* 47 (31.3) | *Total:* 0 | N.a. | | | | |
| Fawzy 2015,  *Interac Cardiovasc Thorac Surg [57]* | Multicenter randomized prospective | 200 | CABG (200) | Posterior pericardiotomy | 1. POD 3 2. POD 5 3. Before discharge | S^‡^  M  L | | | -  -  - | | S + M combined:  65 (32.5)  3 (1.5)  *Total:* 68 (34%) | 0  0  3 (100)  *Total:* 3 (4.7) | No timing described | | | | |
| Ikaheimo 1988,  *Am Heart J [22]* | Prospective cohort | 150 | CABG (100)  Valve(s) (50; AVR (35), MVR (8), valve x2 (2), valve x3 (3)) | Warfarin vs  aspirin-dipyridamol in CABG group | 1. POD 7 2. POD 14 | S  M  L | <10 posterior  >10 posterior  >10 posterior and  anterior | | | | 72 (48.0)  M + L combined:  43 (28.7)  *Total:* 115 (76.7) | -  -  -  *Total:* 1 (0.9) | POD 5 | | 1 | | |
| Inan 2011,  *J Thorac Cardiovasc Surg [15]* | Prospective, randomized | 85 | Aorta (85; Bentall (63), VSRR (7), SCAR (15)) | perioperative administration indomethacin | 1. POD 1 2. POD 7 3. POD 14 4. POD 42 | No PPE sizes described | | | | | *Total*: 9 (10.6) | *Total:* 9 (100)** | POD 1  POD 7  POD 14  POD 42 | | 0  2  3  4 | | |
| Kuralay 1999,  *J Thorac Cardiovasc Surg [58]* | Prospective randomized | 200 | CABG (200) | Posterior pericardiotomy | 1. POD 3-10 2. Before discharge 3. 1 mo after discharge 4. 3 mo after discharge 5. 6 mo after discharge 6. 12 mo after discharge | No PPE sizes described | | | | | *Total:* 76 (38.0) | *Total:* 10 (13.2) | POD <30  POD >30 | | 0  10 | | |
| Luo 2004,  *Am J Cardiol [21]* | Prospective observational | 200 | CABG (97)  Valve(s) (67; AVR±MVR (35), MVR/P (34))  CABG + Valve (23)  Aorta (10); Other (3) | No | 1. POD 3 (if PPE was present, the following were also performed:) 2. POD 4 3. POD 5 4. POD 6 | S‡  M  L | | | -  -  - | | 39 (19.5)  M + L combined:  4 (2.0)  *Total:* 43 (21.5) | -  -  -  *Total:* 4 (9.3) | POD 3  POD 4-6 | | 2  2 | | |
| Malgerud 2020,  *Scand Cardiovasc J [17]* | Prospective randomized | 100 | Valve(s) (all AVR: mechanical 29, biological 71; + 14 concomitant aorta) | Active clearance drain | 1. POD 3-5 | S  M  L | <10  10-20  >20 | | | | 31  22  19  *Total:* 72 | *Total: 0* | N.a. | | | | |
| Mangileva 2021,  *Kardiologiia [14]* | Prospective non-controlled trial | 89 | CABG (53)  Valve(s) (36; AVR (22), MVR (12), valvuloplasty (2)) | In patients with PPS (n=53): postoperative ibuprofen vs prednisolone | 1. POD 20 | No PPE sizes described | | | | | *Total:* 34 (38.2) | No reintervention incidence described | N.a. | | | | |
| Meurin 2004,  *CHEST [8]* | Retrospective cohort | 1277 | CABG (856)  Valve(s) (421) | No | 1. POD 20 2. POD 30 | S  M1  M2  L | <10  10-14  >14 | | | <10  10-14  15-19  >19 | 176 (13.8)  80 (6.3)  20 (1.6)  7 (0.6)  *Total:* 210 (16.4) | 0  5 (6.2)  3 (15.0)  4 (57.1)  *Total:* 12 (5.7) | POD 20  POD 30 | | 0  12 | | |
| Pepi 1994,  *Br Heart J [10]* | Prospective consecutive | 803 | CABG (430)  Valve(s) (330; AVR 158 + MVR 129 +  AVR + MVR 43)  Other (43) | No | 1. POD 8 2. POD 14 3. POD 25 | S  M  L | <10  10-19  >20 | | | <5  5-9  >10 | 341 (42.5)  142 (17.7)  15 (1.9)  *Total:* 498 (62.0) | 0  0  15 (100)**^†^**  *Total:* 15 (3.0) | POD 8  POD 14 | | 6  9 | | |
| Refat 2018,  *J Egyp Soc Cardiothorac Surg [38]* | Prospective randomized | 300 | CABG (180)  Valve(s) (120)  CABG + Valve (15) | Retrocardiac drainage (vs single anterior tube) | 1. POD 1 2. POD 7 3. First control after discharge 4. POD 90 | No PPE sizes described | | | | | *Total:* 24 (8.0) | *Total:* 3 (12.5) | POD ≥7 | | 3 | | |
| Shvartz 2022,  *J Cardiovasc Dev Dis [50]* | Prospective double-blind randomized placebo-controlled | 240 | CABG (180)  Valve(s) (40)  CABG + Valve (20) | Perioperative administration Colchicine | 1. POD 3 2. POD 5 | No PPE sizes described | | | | | *Total:* 51 (21.3) | *Total:* 0 | N.a. | | | | |
| Smulders 1989,  *Ann Thorac Surg [18]* | Prospective randomized | 100 | CABG (83)  Valve(s) (13; AVR (10), MVR (3))  CABG + Valve (4) | Drain duration 24 vs 48 hours | 1. POD 6 | S  M  L | | Slight/thin, below  AV-groove  Uniform PPE,  posterior + apical  Heavy PPE apical, posterior and lateral | | | 11 (11.0)  M + L combined:  22 (22.0)  *Total:* 55 (55.0) | *Total:* 0 | N.a. | | | | |
| Stevenson 1984,  *Am J Cardiol [59]* | Prospective observational | 39 | CABG (24)  Valve(s) (9)  Congenital^⁂^ (6) | No | 1. POD 4 2. POD 10 | S  M  L | | PPE posterior and  Below AV-groove  More uniform PPE  Apical + lateral and  Posteromedial | | | 11 (28.2)  M + L combined:  22 (56.4)  *Total:* 33 (84.6) | 0  0  1 (4.6)  *Total:* 1 (84.6) | No timing described | | | | |
| Tomic 2020,  *Open Access Maced J Med Sci [19]* | Retrospective case-control | 1929 | CABG (1929) | LIMA-LAD vs VSM-LAD  (no intervention, but retrospective analysis) | 1. POD 5 | Min  S  M  L | ≤5  6-10  11-15  >15 | | | | 701 (36.3)  241 (12.4)  152 (7.9)  125 (6.4)  *Total:* 1219 (63.1) | No reintervention incidence described | N.a. | | | | |
| Weitzman 1984,  *Circulation [7]* | Prospective consecutive | 122 | CABG (98)  AVR (13)  CABG + AVR (6)  AVR + MVR (3)  CABG + MVR (1)  Other (3) | No | 1. POD 2 2. POD 5 3. POD 10 4. POD 20-50 | S  M  L | 1-9  10-19  ≥20 | | | S  M  L | 57 (46.7)  45 (36.9)  12 (9.8)  Total: 122 (93.4) | 0  1 (2.2)  0  *Total:* 1 (0.8) | POD 3 | | 1 | | |
| You 2016,  *PLoS One [4]* | Retrospective consecutive | 556 | Valve(s) (± CABG, MAZE, aorta) | No | 1. TTE1 on POD 5 2. Additional follow-up TTE based on clinical decision | S  M  L | <10  10-14  ≥15 | | | 10-14  15-19  ≥20 | 17 (3.1)  7 (1.3)  9 (1.6)  Total: 33 (5.9) | 5 (29.4)  0  5 (55.6)  Total: 10 (30.3)  +14**^#^** | POD <30  POD >30 | | 17  7 | | |

**Table 1.** Table with all included studies with corresponding study characteristics, moments of echocardiography examinations, measured PPE sizes, incidence of PPE and PPE-related reinterventions, and timing of reintervention.
AVR: aortic valve replacement; CABG: coronary artery bypass grafting; L: large; LAD: left anterior descending coronary artery; LIMA: left internal mammary artery; LV: left ventricle; M: moderate; Min: minimal; MVR: mitral valve replacement; MVP: mitral valve plasty; N.a.: not applicable; POD: postoperative day; PPE: postoperative pericardial effusion; S: small; SCAR: supracoronary ascending aorta replacement; TTE: transthoracic echocardiogram; VL: very large.

*Any effusion (also physiological or no apparent effusion) was counted towards the <10 mm effusion size group.
**The study did only noted PPE requiring reintervention (yet described routine echocardiography)
^⁂^all patients undergoing congenital surgery were adults (e.g. septal defects) ^#^study described 14 reinterventions in patients with no apparent PPE (not reported if any cloths were seen on TTE)
^‡^No PPE sizes are described in the methods, but mild, moderate, and large effusion sizes are used in results
^†^of all 15 large effusion reinterventions, 7 were initially moderate but progressed to large, and 2 were large effusions that were initially managed conservatively but persisted.
**^✝^** References numbered ≥ 44 are in the supplement references as those studies are not further mentioned in the review text.
